# Metabolic disease variants rewire gene regulation through disruption of DNA G-quadruplex structures

**DOI:** 10.1186/s13059-026-04147-2

**Published:** 2026-06-10

**Authors:** Angelika Lahnsteiner, Victoria Ellmer, Esther Schönauer, Julia Huber, Matej Lexa, Markus Wiederstein, Angela Risch

**Affiliations:** 1 Division of Cancer (Epi-)Genetics, Department of Biosciences and Medical Biology, University of Salzburg, Salzburg, 5020 Austria; 2 Center for Tumor Biology and Immunology (CTBI), University of Salzburg, Salzburg, 5020 Austria; 3 Cancer Cluster Salzburg, Salzburg, 5020 Austria; 4 Division of Structural Biology, Department of Biosciences and Medical Biology, University of Salzburg, Salzburg, 5020 Austria; 5 Faculty of Informatics, Masaryk University, Brno, 60200 Czech Republic; 6 Division of Protein Bioinformatics, Department of Biosciences and Medical Biology, University of Salzburg, Salzburg, 5020 Austria

**Keywords:** G-quadruplex, Disease-associated SNVs, Metabolic disease, Cancer risk, Transcription factors, Alternative promoters, PDAL-seq

## Abstract

**Background:**

The global prevalence of metabolic diseases (MetDs) continues to rise and is associated with an increased cancer risk. Genome-wide association studies have identified numerous single nucleotide variants (SNVs) linked to MetDs, some within genes also implicated in tumorigenesis. G-quadruplexes (G4s) are non-canonical DNA secondary structures that regulate gene expression in diverse and context dependent ways. Variants affecting G4 structure may alter transcriptional efficiency. Notably, disease-associated variants are frequently located within or near regulatory elements and may overlap with cancer-associated alternative promoters.

**Results:**

We systematically assess the overlap between MetD-associated SNVs and G4 motifs, and evaluate their effects on G4 stability, topology, and their potential to modulate the regulatory activity of G4s in alternative promoters. Approximately 0.9% to 1.6% of MetD-associated SNVs are located within G4 motifs (G4-SNVs), depending on the prediction tool. Effect alleles, those associated with risk or protection against MetDs, generally reduce G4 stability, regardless of their direction of association. Several G4-SNVs are mapped to cancer-associated alternative promoters, including the destabilizing *MICB* rs2855804 C/T variant and the stabilizing *PLA2G6* rs2277844 G/A variants. In vivo G4 formation is confirmed by permanganate/S1 nuclease footprinting coupled with sequencing, while circular dichroism spectroscopy reveals allele-specific changes in G4 topology and stability. Integration of Hi-C data, histone modifications, transcription factor binding, and luciferase reporter assays further support their regulatory impact.

**Conclusions:**

Although G4-SNVs are unlikely to be the sole disease drivers, they significantly influence transcriptional regulation, potentially contributing to allele-specific gene expression in MetD patients and their link to increased cancer risk.

**Supplementary Information:**

The online version contains supplementary material available at 10.1186/s13059-026-04147-2.

## Background

G-quadruplexes (G4s) are non-canonical (non-B) DNA structures increasingly recognized as dynamic regulatory elements that influence a wide range of key biological processes, including recombination [1, 2], DNA repair [3] and transcription [4, 5]. G4s form in guanine-rich regions satisfying the motif G_≥3_N_x_G_≥3_N_x_G_≥3_N_x_G_≥3_, where N represents any base and x typically denotes a 1–7 bp DNA linker [6, 7], albeit more relaxed patterns are also known [8]. G4s are held together by Hoogsteen hydrogen bonds [9] and are stabilized via monovalent cations, typically in the order of K^+^ > Na^+^ > Li^+^ [10, 11]. While stretches of consecutive guanines assemble into the G4 stems, the intervening bases form connecting loops, giving rise to various topologies such as parallel, antiparallel or hybrid structures [12]. Short loop lengths correlate with higher G4 stability [8, 13, 14]. G4s are not randomly distributed but enriched in nucleosome-depleted regions and regulatory elements, such as promoters [15–18], enhancers [17, 19] and within specific transposable elements and tandemly repeated sequences [20, 21], which have been hypothesized to serve as vehicles for their multiplication in the genome [22], possibly prior to their fixation.

The formation, topology and stability of G4s are determined by several factors including pH, cation concentrations, the presence of helicases [23], or their sequence motifs, where nucleotide changes within the motif can significantly alter structural integrity [24]. As such, SNVs located in G4 motifs can modulate structural properties of G4s and, in turn, influence gene expression patterns [24–26].

Genome-wide association studies (GWAS) have identified thousands of SNVs associated with a wide range of human traits and diseases, including MetDs like the metabolic syndrome (MetS) [27], a condition characterized by visceral obesity, disturbed glucose and fatty acid metabolism, insulin resistance and hypertension [28]. MetS is often underdiagnosed and can persist for years before detection. It is associated with an increased risk for metabolic-associated fatty liver disease (MAFLD) and type II diabetes [28, 29], both of which further increase the likelihood of cancer development [30]. However, the molecular mechanisms by which these genetic variants exert their effects remain unclear.

Recent studies have shown that alternative (or cryptic) promoter elements are differentially regulated across various tumor types, giving rise to aberrant – often disease causing – isoforms [31–33]. Given the regulatory potential of G4 structures, there is growing interest in whether MetD-associated SNVs overlap with G4 motifs, how they affect G4 stability or topology, and whether these elements occur in cancer-associated alternative promoters and consequently influence transcription patterns in an allele-specific manner. We hypothesize that SNVs within G4-forming sequences (G4-SNVs) can alter structural properties of G4s, thereby affecting gene expression, potentially within alternative promoter elements of genes involved in tumor progression and immune system function, which may help to partially explain the elevated cancer risk observed in MetD patients.

To address this, we aimed to identify SNVs overlapping with G4 motifs predicted by pqsfinder [34] and G4Hunter [35, 36] tools, assess their impact on G4 stability and topology, analyze the overlap with disease-associated alternative promoters and explore their functional relevance in metabolic diseases and hepatocellular carcinoma (HCC). Two identified G4-SNVs were investigated in more detail: a G4-destabilizing variant in MHC class I polypeptide–related sequence B (*MICB*), a surface antigen associated with tumor immune evasion [37], and a G4-stabilizing variant in the tumor-promoting gene patatin-like phospholipase domain-containing protein 9 (*PLA2G6*) [38]. Both variants have in common that they alter structural properties of G4s and are associated with allele-specific expression patterns.

Our findings indicate that G4-SNVs show a significantly higher rate of G > A substitutions compared to SNVs outside of G4 motifs and can alter transcription factor binding sites in an allele-specific manner. Furthermore, they drive genotype-specific transcription patterns by influencing G4 stability and topology in two alternative promoter elements within *MICB* and *PLA2G6*, thereby providing a mechanistic link between genetic predisposition and changes in gene expression that may contribute to disease development and progression.

## Results

### Metabolic disease risk SNVs overlap with G4 motifs

To investigate whether SNVs associated with MetS – a form of metabolic disease – influence the stability or topology of G4 structures and whether certain candidates overlap with cancer-associated alternative promoters, we analyzed SNVs identified from a GWAS including 1,252,787 individuals of European ancestry [27]. We included all SNVs with a Benjamini Hochberg (BH) corrected *p* < 0.05, regardless of whether the effect alleles were positively (risk alleles) or negatively associated (protective alleles) with the disease, as determined by the direction of the beta value (β) in the GWAS. This resulted in a dataset of 618,106 significant MetD-associated SNVs, which were further investigated.

G4 structures were predicted using pqsfinder [34] and G4Hunter [35, 36] as described in the methods section. Pqsfinder detects G4 structures by identifying sequences that resemble the G4 motif G_≥3_N_x_G_≥3_N_x_G_≥3_N_x_G_≥3_ but allowing for imperfections like bulges (intervening nucleotides in a stem, e.g. GGACG) or mismatches within the G-tract. In contrast, G4Hunter uses a sliding window approach to score regions based on G-content and G-skewness, identifying sequences with a high potential to form G4 structures regardless of specific motif patterns. These tools were chosen due to their recent application in studies using the new telomere-to-telomere (T2T) genome assemblies from human and other great apes by Mohanty and colleagues [39], as well as their best overall performance in in vitro datasets [40]. The predicted G4 motif datasets were then intersected with the 618,106 significant MetD-associated SNVs.

**Fig. 1:**
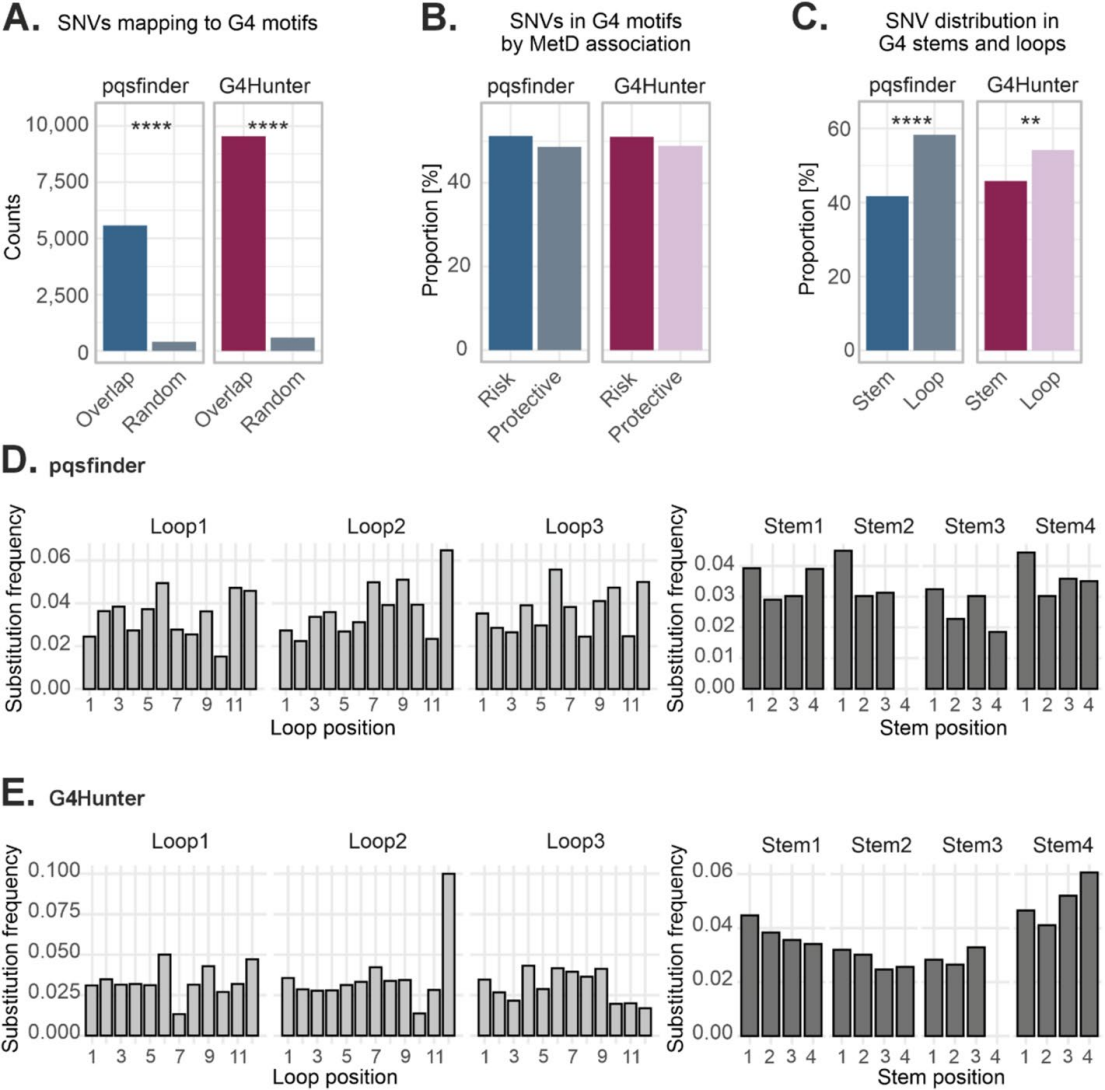
MetD-associated SNVs are located in G4 motifs. **A** SNVs are significantly enriched in G4 motifs predicted by pqsfinder [34] and G4Hunter [35, 36]. Permutation tests with 1,000 iterations using a randomized SNV dataset demonstrated that their occurrence was unlikely to be due to chance. **B** Risk SNVs showed a slightly higher enrichment in G4 motifs compared to protective SNVs, albeit the difference was not significant (Fisher’s exact test). **C** G4-SNVs were more frequently located in loop than in stem regions, regardless of the prediction algorithm. Proportions [%] reflect substitutions detected per total canonical motifs. Significance was determined using a Benjamini Hochberg (BH) corrected Fisher’s exact test. **D-E** Detected substitution frequencies per bp are shown for each individual position within loops (1–3) and stems (1–4) in pqsfinder- **D** and G4Hunter- **E** predicted canonical G4 motifs. Left panels display the substitution frequencies per loop position of loop 1–3, whereas right panels show the total number of substitutions per stem position of stem 1–4. ****p < 2e-16, *** *p* < 0.001, and ** *p* < 0.01

Our analysis resulted in 5,576 (0.90%) SNVs overlapping G4 motifs (from now on called G4-SNVs) predicted by pqsfinder (Additional file 1: Table S1) and 9,562 (1.55%) G4-SNVs were observed when predicting G4-motifs with G4Hunter (Fig. 1A, Table 1, Additional file 1: Table S2). While 26.4% of G4-SNVs were shared between both tools, 20.2% were unique for pqsfinder and 53.4% for G4Hunter (Additional file 2: Fig. S1, Additional file 1: Table S3), reflecting differences in the underlying G4 prediction algorithms. The low number of detected G4-SNVs can be partly explained by the elevated AT content within ± 25 bp windows centered around the MetD-associated SNVs (Additional file 2: Fig. S2). Furthermore, we identified 48 G4 motifs detected by both tools, which contained at least two SNVs per motif (Additional file 1: Tables S4-S5). Analysis of the association between risk or protective SNVs in G4s showed a slight enrichment of risk SNVs within G4 regions for both tools, although this enrichment did not reach statistical significance (Fig. 1B).

**Table 1:** SNVs overlapping with G4s predicted by different tools. SNVs associated with metabolic diseases identified by a GWAS [27] were intersected with pqsfinder [34] and G4Hunter [35, 36] predicted G4 motifs. The total number of overlapping SNVs is shown, including unique and shared variants between the two G4 prediction tools, as well as the number of risk and protective G4-SNVs

Tool	Total SNVs	Total G4 motifs	SNVs within G4 motifs	Unique	Shared	Risk	Protective	Canonical motifs	Shared canonical motifs
pqsfinder	618,106	1,352,359	5,576 (0.90%)	2,420	3,156	2,860	2,716	1,756	801
G4Hunter	618,106	2,446,251	9,562 (1.55%)	6,387		4,880	4,682	1,096	

To assess whether these overlaps occurred by chance, we generated a randomized SNV set matching the chromosomal distribution, as well as the GC content of the identified SNVs and overlapped it with the G4 motifs predicted by pqsfinder and G4Hunter. Only 7.73% of 5,576 randomized SNVs intersected with pqsfinder-predicted G4 motifs and 6.54% of 9,562 with G4Hunter-predicted motifs (*p* < 0.001, permutation test with 1,000 iterations), indicating that the observed overlaps were unlikely to occur randomly (Fig. 1A, Additional file 1: Table S6). Furthermore, we performed a similar analysis by randomly shuffling the 618,106 significant MetD-associated SNVs across the genome while correcting for the SNV distribution across individual chromosomes and GC content. This analysis revealed a slight but significant enrichment of SNVs within G4 regions predicted by both tools (Additional file 2: Fig. S3, Additional file 1: Table S6). Taken together, these results indicate that MetD-associated SNVs do not occur in G4s solely by random chance or due to GC content but rather suggest that G4 structures may contribute to the increased occurrence of SNVs.

Guiblet et al. [41] reported that G4 loops are more prone for substitutions than stems. Since we focused on only a subset of SNVs – specifically those associated MetDs – we were curious whether we can detect a similar trend in our dataset. Therefore, we selected all canonical G4s allowing loop lengths of up to 12 base pairs (G_≥3_N_1–12_G_≥3_N_1–12_G_≥3_N_1–12_G_≥3_) without bulges from the pqsfinder- and G4Hunter-predicted G4 motifs overlapping with MetD-associated SNVs. Of the total 5,576 G4-SNVs predicted by pqsfinder, 1,756 were identified as canonical motifs and 1,096 of 9,562 motifs for G4Hunter, of which 801 were shared between both tools (Additional file 1: Tables S7–S9). By calculating the proportions of substitutions in stems and loops relative to the total number of detected canonical motifs, both algorithms revealed a significant enrichment of loop substitutions (Fig. 1C, Additional file 1: Table S10). To account for differences in stem and loop length, the number of SNVs was normalized by the total number of analyzed nucleotides of each region (substitutions per base pair), resulting in nearly equal stem and loop substitution frequencies for pqsfinder G4 motifs (Additional file 2: Fig. S4 and Additional file 1: Table S10). For G4Hunter-predicted motifs, the trend was even slightly reversed, with a higher substitution frequency in stems compared to loops.

As not every loop or stem position is affected by similar substitution frequencies [41], we compared individual positions of each loop (loops 1–3) and stem (stems 1–4) independently. We did not observe a clear positional effect with only a slight enrichment of loop substitutions in flanking regions for pqsfinder (Fig. 1D), but not for G4Hunter (Fig. 1E). The increased substitution frequency of position 12 of loop 2 must be interpreted with caution, since there are only a few G4 motifs with 12 nt long loops (average loop length: pqsfinder 5.74 nts, G4Hunter 5.56 nts; Additional file 1: Table S10 and Additional file 2: Fig. S5). Across stem regions, both algorithms indicated slightly overall higher substitution frequencies in stem 1 and 4, compared to stem 2 and 3, albeit more prominent for G4Hunter-predicted G4 motifs. Position 4 of pqsfinder-predicted G4 stem 2 and G4Hunter stem 3 showed no substitutions. Average stem and loop lengths in nucleotides were comparable between pqsfinder- and G4Hunter predicted motifs (Additional file 1: Table S10 and Additional file 2: Fig. S5).

One major predictor of increased substitution frequency is elevated GC content [42]. We assessed the base composition of G4 stems and loops from canonical G4 motifs overlapping with SNVs compared to non-SNV G4 motifs. As control, we used all canonical G4 motifs predicted by both tools, which do not harbor MetD-associated SNVs. Since this number was substantially larger than the number of identified G4-SNVs, we performed random sampling of the canonical control G4 datasets to obtain an equal number of motifs (1,756 for pqsfinder and 1,096 for G4Hunter). This random sampling was repeated 1,000 times to generate a background distribution. Since we defined the stems as tracts with G_≥3_ and did not include bulged G4 motifs, the GC content in stems was equal to 1, and therefore not included in the plot. The first nucleotide position of all three loops consistently showed a reduced GC content compared to subsequent loop positions for G4 motifs harboring an SNV and the background dataset. However, for G4-SNVs, the GC content was even significantly lower at position 1 for loop 3 for pqsfinder and loop 1–3 for G4Hunter compared to the control (Additional file 2: Fig. S6). This observation is consistent with the top motif – G_3_AG_3_AG_3_AG_3_ containing a single-A-nucleotide loop – identified by both algorithms (Additional file 2: Fig. S7). The GC content of loops harboring MetD-associated SNVs was generally lower than that of the control dataset for pqsfinder, which was not detected to the same degree for G4Hunter-predicted G4s (Additional file 2: Fig. S6). The GC content at individual loop positions across loops 1–3 was mostly lower for G4s harboring MetD-associated SNVs compared to control G4s, with 14 of the 36 total positions showing significantly lower GC content according to pqsfinder (Additional file 2: Fig. S6A). In contrast, for G4Hunter-predicted G4s, only 5 of the 36 positions showed a significant difference in GC content (Additional file 2: Fig. S6B). Across positions 2–12, the GC content ranged from approximately 45–68%, with less variation in loops 2 and 3 than in loop 1. For both prediction tools, GC content tended to decrease towards the last loop position. Since the GC content did not differ significantly between the control and MetD-associated SNV G4 datasets at most loop positions — and was overall lower than in the GC-rich stems — we can exclude the possibility that the slightly higher number of observed loop substitutions (at least as relative counts) are solely attributable to differences in GC content, rather than to the G4 as structural element (e.g., barrier for polymerases).

### MetD-associated SNVs show distinct substitution patterns and lead to significantly reduced G4 stability

**Fig. 2:**
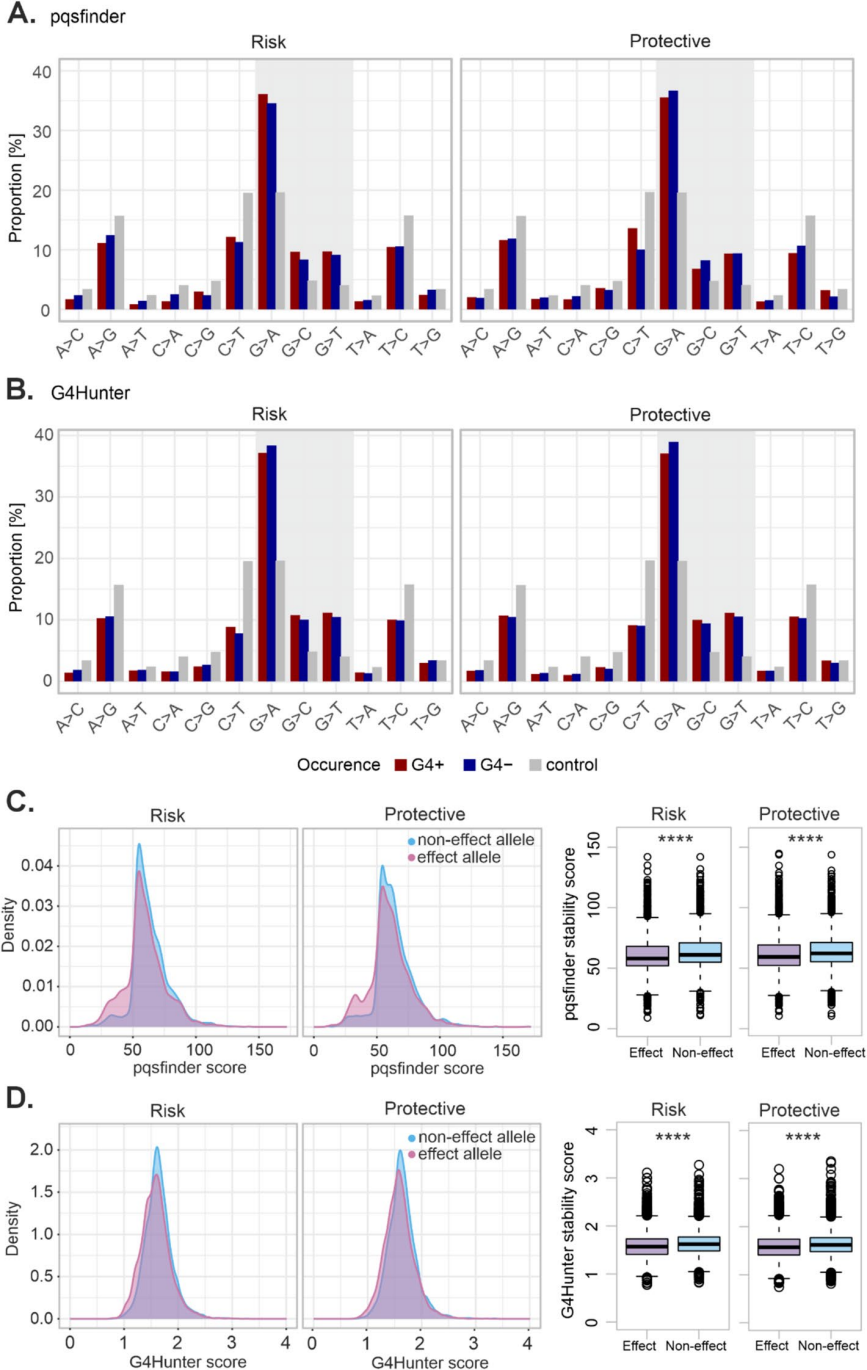
MetD-associated SNVs and their effect on G4 stability. **A**, **B** Analysis of nucleotide substitution patterns in variants overlapping G4s predicted by pqsfinder **A** or G4Hunter **B**, separated into risk or protective MetD-associated G4-SNVs. Substitution direction was further stratified by G4 strand orientation (plus or minus strand occurrence of the G4 motif) and compared to variants outside of G4s from the same dataset. For control SNVs, substitution proportions were calculated using the plus strand only. The grey-shaded regions highlight substitutions that are highly enriched within G4 motifs. Statistical analysis using a BH adjusted Fisher’s exact test is reported in Additional file 1: Table S11. **C**, **D** Density plots (left panel) and boxplots (right panel) showing G4 stability scores for G4 motifs containing the effect allele (red) versus the non-effect allele (blue) for risk and protective SNVs for pqsfinder **C** and G4Hunter **D** Stability scores of G4-SNVs were reduced in the presence of effect alleles for both risk and protective variants. Statistical significance was assessed using Wilcoxon rank sum tests. ****p < 2e-16

To assess differences in the direction of nucleotide substitutions between G4-SNVs and non-G4-SNVs (controls), substitutions were classified by type (e.g., A > C, G > A) and stratified by risk versus protective alleles, as well as by the occurrence of the G4 motif on the plus (G4^+^), or on the minus strand (G4^−^). Control SNVs were selected from genomic regions with similar GC content and were depicted only for the plus strand. Both G4^+^- and G4^−^-SNVs exhibited significantly increased G > A substitutions compared to control SNVs, suggesting that this enrichment is dependent on G4 structures but independent of strand orientation (Fig. 2A. pqsfinder and B. G4Hunter). This pattern was consistently observed for both pqsfinder- and G4Hunter-predicted G4 motifs, and for risk and protective alleles. In contrast, other substitution types, such as A > C, T > C, or C > T, were evenly distributed or enriched in control regions. Statistical analysis can be found in Additional file 1: Table S11.

For both algorithms, the overall differences between strands were modest, indicating largely strand-independent substitution patterns within G4 motifs (Additional file 2: Fig S8). For pqsfinder, risk alleles tend to show slightly more C > G/T, and G > A/C/T on the G4^+^ strand. In protective variants, most substitution types were slightly more frequent on the G4^−^ strand or symmetrically distributed, except for a C > G/T and T > G excess on the plus strand. Strand-specific substitution direction was less clear for G4Hunter-predicted G4-SNVs, with only a few substitution directions being identical with pqsfinder G4-SNVs, at least among the risk SNVs. Differences between G4^+^ and G4^−^-SNV occurrence were not significant (Additional file 1: Table S11).

The distribution of G4-SNVs within ± 1,000 kb of the transcription start site (TSS) showed a strong enrichment near the TSS and similar strand-specific distributions with no significant difference for both tools (Additional file 2: Fig. S9).

Given the elevated substitution frequency in G4 motifs, we analyzed the effect of MetD-associated SNVs on G4 stability using pqsfinder and G4Hunter scores. We compared the scores of the effect alleles to those of the non-effect alleles separately for risk and protective SNVs. Pqsfinder- and G4Hunter-predicted G4-SNVs exhibited a significant reduction in G4 stability for the effect alleles compared to the non-effect alleles as depicted in density plots and boxplots, independent of whether the variants were classified as risk or protective SNVs (Fig. 2C. pqsfinder and D. G4Hunter).

Taken together, these results demonstrate that G4-associated SNVs exhibit a distinct bias toward G > A substitutions, largely independent of strand orientation or disease association, but with an impact on the overall G4 stability scores. No significant difference was observed in their distribution between the template and non-template strands around the TSS.

### G4-SNVs are enriched in intronic regions and affect the transcription factor binding landscape

**Fig. 3:**
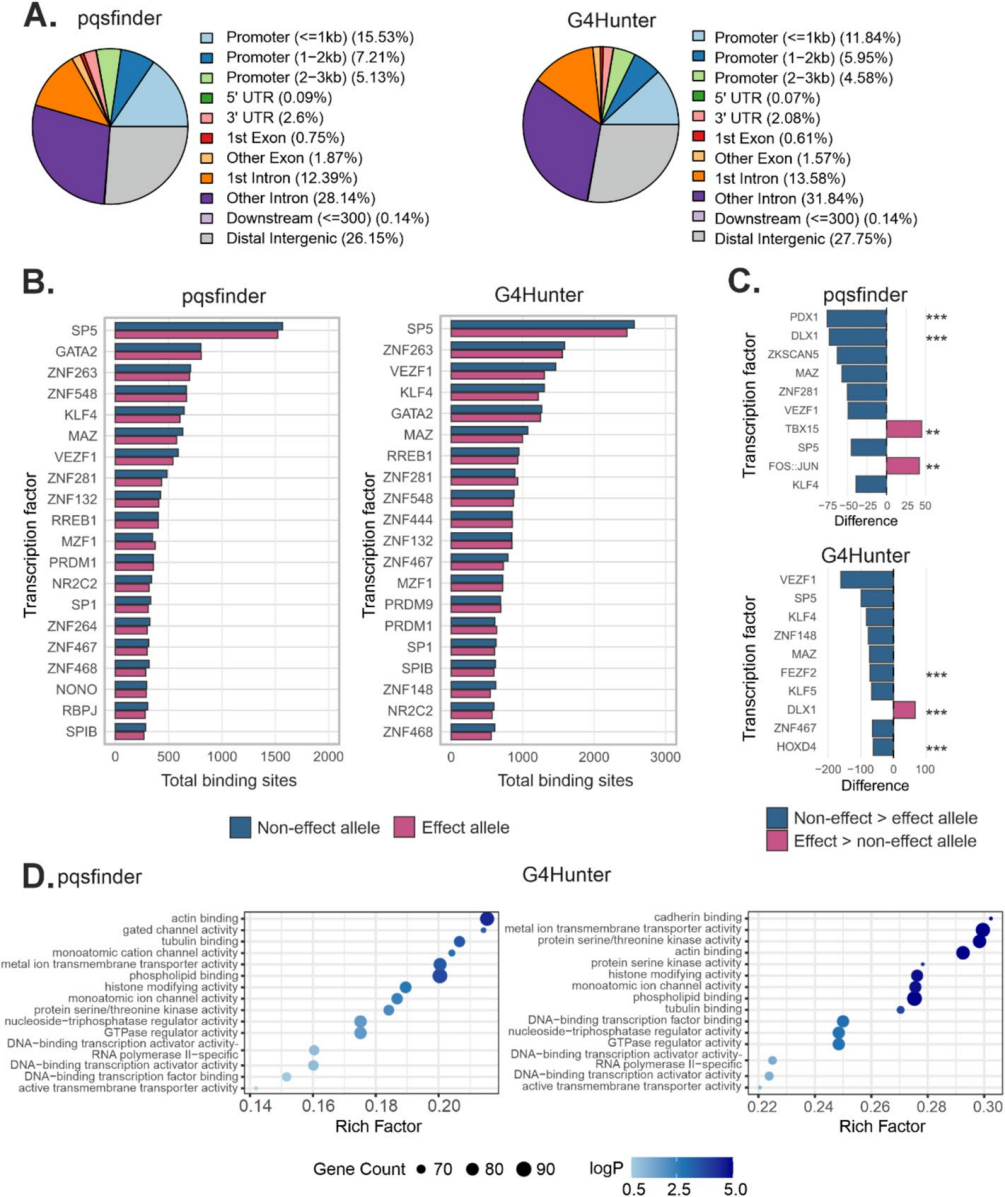
Annotation of G4-SNVs. **A** Distribution of pqsfinder- and G4Hunter-predicted G4-SNVs across genomic features. **B** Overlap of G4-SNVs with motifs for transcription factors from the JASPAR database [43], shown separately for G4 motifs harboring the effect (violet) or non-effect allele (blue). **C** Top 10 transcription factors showing the largest differences for their binding sites between G4 motifs containing either the effect or non-effect alleles of SNVs within pqsfinder- (top) and G4Hunter predicted G4 motifs (bottom). Blue bars indicate higher prevalence in the non-effect allele, while violet bars indicate higher prevalence in effect alleles. Significant changes in TF binding sites are indicated on the right side (BH-adjusted Fisher’s exact test). Positive values indicate TFs enriched for the effect alleles, whereas negative values represent enrichment for the non-effect alleles. **D** Shows Gene Ontology (GO) term analysis for pqsfinder (left) and G4Hunter (right). The color scheme represents the –log₁₀ of the BH-adjusted *p*-value, while the size of each circle indicates the number of genes associated with the corresponding GO term. The Rich Factor represents the ratio of the number of genes annotated to a given GO term to the total number of genes annotated in that category, reflecting the degree of enrichment for that term. *** *p* < 0.001 and ** *p* < 0.01

Annotation of the identified G4-SNVs (5,576 for pqsfinder and 9,562 for G4Hunter) revealed that most variants mapped to intronic regions, followed by promoter and distal intergenic regions (Fig. 3A). Although pqsfinder and G4Hunter predominantly predict G4 motifs in promoter regions, SNVs are highly abundant in intronic and distal intergenic regions, explaining the overall shift from promoter to intronic localization of G4-SNVs (Additional file 2: Fig. S10).

Given the role of G4s as transcription factor (TF) binding hubs [5], we assessed their overlap with known TF binding sites (TFBS) obtained from the JASPAR database [43] and compared the effect of the different G4-SNV alleles on the TFBS. Both tools resulted in similar patterns, with speciation protein 5 (SP5) emerging as the top hit, as well as a substantial enrichment of binding sites for zinc-finger (ZnF) proteins, with 17 ZnF proteins among the top 20 TFs (Fig. 3B). Consistent with previous reports, the G4-binders SP1 [4] and Myc-associated zinc finger protein (MAZ) [44] ranked amongst the top associated TFs. In general, the number of hits was greater for G4Hunter predicted G4 motifs compared to pqsfinder G4 motifs.

Analysis of the effect allele’s influence on TF occurrence revealed that, among the top 10 TFs, effect alleles were mostly associated with decreased TFBS overlap (Fig. 3C). Although the two prediction tools reported slightly different sets of TFs influenced by G4-SNVs, several TF binding sites – for VEZF1, SP5, KLF4, and MAZ – were consistently among the most affected. Notably, DLX1 showed higher enrichment at the non-effect allele in pqsfinder-G4 predictions, but this pattern shifted toward the effect allele in the G4Hunter dataset (Fig. 3C). Significant alterations in TFBS occurrence were observed for PDX1, DLX1, TBX15 and FOS::JUN for pqsfinder G4-SNVs and for FEZF2, DLX1 and HOXD4 for G4Hunter G4-SNVs. Of note, the top altered TFBS among pqsfinder-predicted G4-SNVs overlapped with pancreatic and duodenal homeobox 1 (PDX1), a transcription factor crucial for the maturation of insulin-producing pancreatic β-cells and implicated in the development of various forms of diabetes [45].

Gene Ontology (GO) analysis of both G4-SNV datasets revealed a substantial overlap in enriched terms, particularly those related to transcription factor binding, histone-modifying activity, and cytoskeletal interactions such as actin and tubulin binding (Fig. 3D), consistent with the observed changes in TF binding sites associated with G4-SNVs. Moreover, terms associated with calcium metabolism – including cadherin binding – also emerged. This is of particular interest, as MetDs are characterized by impaired calcium signaling [46].

Together, these findings indicate that G4-SNVs are preferentially located in intronic regions, where they may contribute to altered transcription factor binding by changing their binding sequences. Underlined by GO terms linked to transcription factor binding further suggests a potential mechanistic link between G4-SNVs and dysregulated gene expression in metabolic disorders.

### G4-SNVs are located in cancer-associated alternative promoter elements

Metabolic disorders are associated with an increased risk for specific cancers, including liver [47], lung [48, 49] or pancreas [50]. Recent research has shown that both MetDs and cancer are accompanied by alterations in transcriptional patterns across multiple tissues [51–53]. Importantly, transcription does not always initiate from canonical promoters; instead, it can also start from alternative (or cryptic) promoter elements – a phenomenon observed across many tumor types [31, 32]. Such alternative initiation can generate transcript isoforms, some of which may contribute to disease progression. Activation of alternative promoters can result from loss of DNA methylation, or also from SNVs - both mechanisms which potentially alter G4 stability or topology, thereby modulating their promoter activity and regulatory potential.

We found that 194 pqsfinder- and 295 G4Hunter-predicted G4-SNVs are located near alternative promoters (Additional file 1: Tables S14-S15). Notably, 98 alternative promoters were shared between both tools. To prioritize functionally relevant candidates, we filtered the 98 shared G4-SNVs for those that induced stronger predicted changes in the G4 stability between effect allele and non-effect allele by filtering for a |Δpqsfinder score|= effect allele score – non-effect allele score > 10 and |ΔG4Hunter score|> 0.1, resulting in 52 high-confidence G4-SNVs at cancer-associated alternative promoters (Additional file 1: Table S16). These thresholds were chosen based on the distributions of ΔG4 stability scores (Additional file 2: Fig. S11). While most SNVs had only minor effects on stability scores (high densities at |Δscore|= 0), a noticeable inflection in the distributions was observed at approximately ± 0.1 for G4Hunter scores and, less distinctly, at around 2.5–10 for pqsfinder scores. To focus on those G4-SNVs with detectable effects on G4 stabilities, we have chosen these two thresholds (|Δpqsfinder score|> 10 and |ΔG4Hunter score|> 0.1) for subsequent analyses but keeping in mind that there are plenty of G4s with MetD-associated SNVs being affected only by subtle changes in stabilities, probably also affecting transcription efficiencies.

We assessed whether these SNVs showed genotype-dependent specific gene expression patterns using data from the Genotype-Tissue Expression (GTEx) project [54] across three tissues: liver, lung, and pancreas (Additional file 1: Table S17). Eleven G4-SNVs displayed genotype-specific expression in at least one tissue (Table 2). Among those, three showed a highly significant association with MetDs, with a BH-adjusted *p* < 5 × 10⁻⁸ – a threshold which is commonly used to determine if a variant is significantly associated with a trait [55] – with two G4-SNV identifying as G4-destabilizing and one as a stabilizing variant. To investigate their function in more detail, we focused on those with greater overlap with predicted TF binding sites. This led us to MHC class I polypeptide-related sequence B (*MICB*) and phospholipase A2 group VI (*PLA2G6*).

**Table 2:** G4-SNVs overlapping with annotated alternative promoter elements and differential expression in liver, lung and pancreas identified by Demircioglou et al*.* [32] sorted by GWAS Benjamini Hochberg (BH) adjusted *p*-value. ΔG4Hunter or Δpqsfinder scores represent the difference in predicted G4 stability between the effect and non-effect alleles. Negative values indicate destabilizing variants, while positive values indicate stabilizing variants. The beta value reflects the direction of association with metabolic disorders, with negative values indicating protective SNVs and positive values indicating risk-associated SNVs

SNV ID	Chr	SNV position	Start G4	End G4	Gene	Transcription factors	Δpqsfinder score	ΔG4Hhunter score	Beta value	BH adj.* p*	Differential expression by genotype
rs4318227	chr16	29,973,517	29,973,502	29,973,520	*TMEM219, TAOK2*	ETV7	−28	−0.21	0.0196	4.45 × 10^–47^	Lung
rs2855804	chr6	31,499,587	31,495,936	31,499,935	*MICB*	KLF5, KLF16, SP8, MAZ, SP9, SP3, SMAD2, KLF1, SP4, KLF14, KLF12, KLF10	−17	−0.13	0.0082	5.91 × 10^–10^	Liver, Lung, Pancreas
rs2277844	chr22	38,181,507	38,181,755	38,182,754	*PLA2G6*	ZBTB6	12	0.29	−0.0072	1.18 × 10^–09^	Lung, Pancreas
rs4319473	chr11	46,681,140	46,681,011	46,681,109	*ARHGAP1*	ELK4, ETV6, FLI1, ERF, ETS1, ETV2, ETV3, ELK1, ZBTB7A, ETS2, PLAGL2, VEZF1, ETV1, GABPA, FEV, ELK3, ETV4, ETV5	−26	−0.66	0.0086	2.76 × 10^–07^	Liver, Pancreas
rs2303516	chr15	41,836,369	41,835,785	41,836,784	*PLA2G4B*	RXRA::VDR, PLAGL2, ZNF320	−13	−0.33	0.0051	4.14 × 10^–05^	Liver, Lung, Pancreas
rs2061690	chr1	154,946,603	154,945,572	154,946,803	*PBXIP1*	PLAG1, NEUROD1, KLF6, HAND2, NEUROG2, TWIST1, ZNF416, ZNF692	−11	−0.20	−0.0045	2.14 × 10^–04^	Lung, Pancreas
rs4925130	chr17	17,904,543	17,898,675	17,907,446	*TOM1L2*	SMAD2	−16	−0.41	0.0041	9.16 × 10^–04^	Lung
rs450208	chr11	2,910,750	2,909,711	2,916,621	*SLC22A18*	GCM2	28	0.35	0.0043	2.93 × 10^–03^	Lung
rs9263745	chr6	31,145,548	31,145,460	31,146,459	*CCHCR1*	ZNF816, PRDM1	−15	−0.40	−0.0040	1.43 × 10^–02^	Lung
rs730291	chr19	5,070,797	5,069,618	5,070,617	*KDM4B*	KLF5, FIGLA, ZEB1, SI1, SI3, ASCL1, TCF12, ZNF148, KLF4, MYOD1, SI2, TCF3, TCF4, PATZ1	−17	−0.39	−0.0033	1.70 × 10^–02^	Liver, Pancreas
rs4783950	chr16	56,677,645	56,677,470	56,681,469	*MT1X*	RBPJ, ZNF93, ZNF189	13	0.30	−0.0029	1.71 × 10^–02^	Lung

**Fig. 4:**
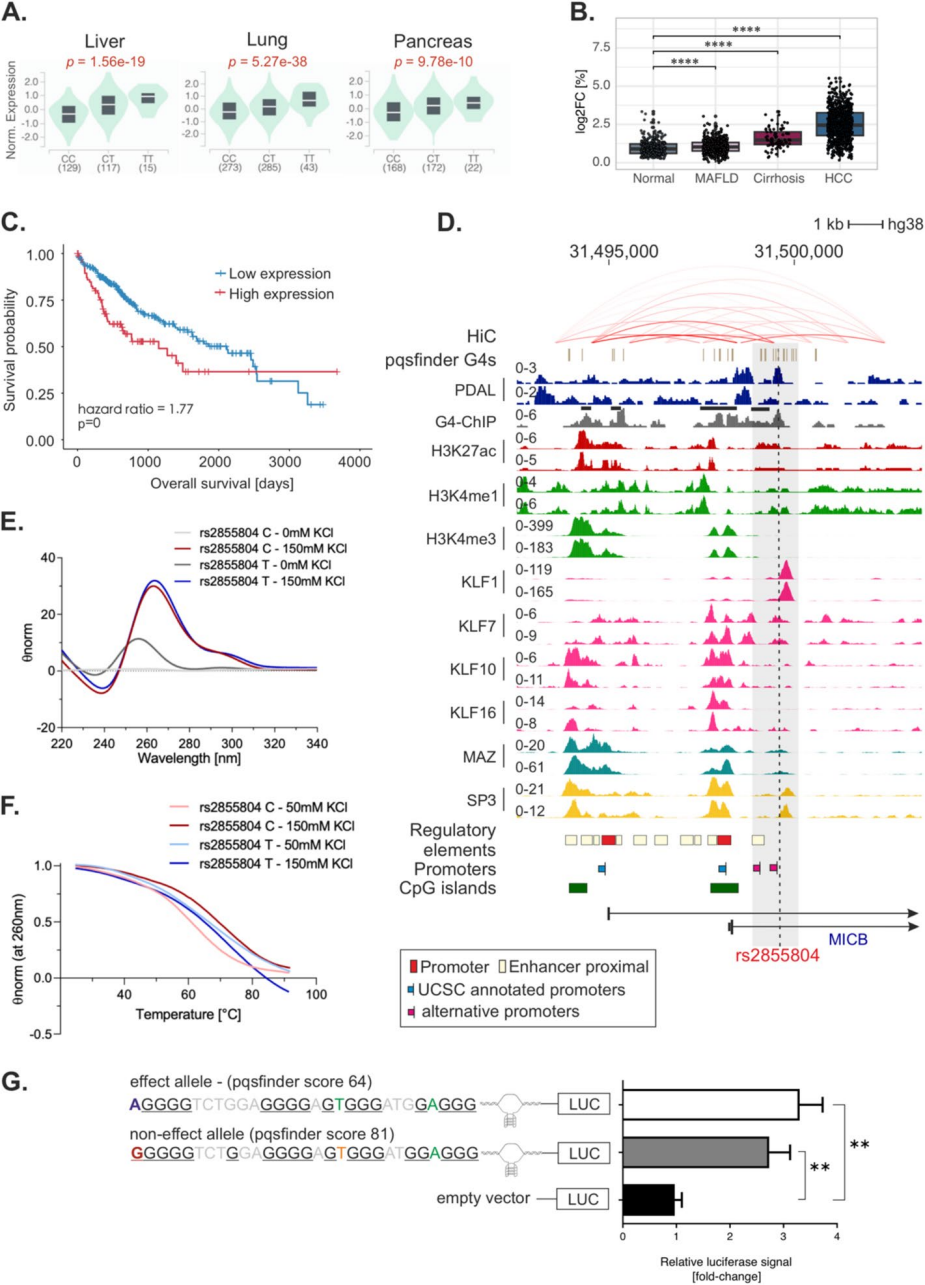
Molecular characterization of the rs2855804 C/T G4-SNV in intron 1 of *MICB*.** A** *MICB* expression correlated with the genotype of the G4-SNV rs2855804 in three different tissues. Data were obtained from the GTEx database [54], and *p*-values were adjusted for multiple testing using false discovery rate (FDR) correction. **B** *MICB* expression patterns increased across different phenotypes from healthy tissue to metabolic-associated fatty liver disease (MAFLD) to hepatocellular carcinoma (HCC) liver tissue. Data obtained from GepLiver database [65]. *P*-values were calculated using Wilcoxon rank sum test with BH adjustment.** C** Elevated *MICB* expression caused significantly lower overall survival of HCC patients. Data were obtained from TCGA [57]. Significance was assessed using a log-rank test. **D** The G4-SNV colocalized with pqsfinder-predicted G4 motifs (beige), PDAL-seq valleys (dark blue), and G4-ChIP-seq peaks (grey) in HEK293 cells. Modest enrichment of H3K27ac (red) and H3K4me1, but not H3K4me3 (green) was observed. KLF1, KLF7, SP3, and to a lesser extent also MAZ binding was detected surrounding this region. Hi-C contacts revealed strong interaction between this locus and the upstream promoter. **E** CD spectra of the G4 motifs containing the effect allele (T) and the non-effect allele (C) demonstrated parallel G4 formation in a K^+^ concentration-dependent manner. **F** CD melting curve analyses were performed with two different buffer conditions containing either 50 mM or 150 mM KCl. Both conditions showed different stabilities of the alleles, with the effect-allele (T) being more stable with 50 mM KCl (ΔTm = 5.7 °C, p = 2.942 × 10^–6^, Welch’s t-test), but less stable with 150 mM KCl compared to the non-effect allele (C, ΔTm = 9.4 °C, p = 0.014, Welch’s t-test). **G** The G4-motif containing either the effect (T, complementary to A in the G4 motif) or the non-effect allele (C, complementary to G in the G4 motif), were cloned upstream of the luciferase gene on the template (non-coding) strand. The underlined bases represent the G-tract positions, while grey bases form the loop regions. Green nucleotides represent bulges and the orange nucleotide a mismatch in the G-tract. The G4-SNV alleles are marked in red (C, complementary to G in the G4 motif) and blue (T, complementary to A in the G4 motif). G4 motifs and their scores were predicted by pqsfinder. Luciferase reporter assays in HEK293 cells revealed a 2.7-fold increase in promoter activity of the G4 including the non-effect allele C compared to the empty vector, while the effect allele T caused a 3.3-fold promoter function (Welch’s t-test). **** *p* < 0.0001 and ** *p* < 0.01

The *MICB* risk SNV rs2855804 C > T (corresponding to a G > A substitution within the G4 motif located on the template or non-coding strand) is associated with a predicted loss in G4 stability. According to the dbSNP database of the National Center for Biotechnology Information (NCBI) the allele frequencies for *MICB* of individuals with European ancestry (sample size 20,916) are C = 0.684/T = 0.316 [56]. Analysis of GTEx data revealed significantly higher *MICB* expression in individuals with the TT genotype (Fig. 4A). Although genotype-specific transcript expression data from MetD patients were not available, *MICB* expression increased progressively across metabolic disease stages, from MAFLD to cirrhosis and hepatocellular carcinoma (HCC, Fig. 4B), supporting its positive association with metabolic disease risk. Survival analysis using data from 361 HCC patients from the *The Cancer Genome Atlas* (TCGA) [57] demonstrated that high *MICB* expression was significantly associated with reduced overall survival (hazard ratio = 1.77, *p* = 0, Fig. 4C). *MICB* is not constitutively expressed in normal tissue, since it acts as “killing signal” [58]. It harbors five annotated alternative promoters, four lie within intron 1 (hg38; chr6: 31,498,678–31,503,303; Additional file 2: Fig. S12). Notably, one of them overlaps the G4-SNV and is located slightly downstream of a putative enhancer region, as defined by the Chromatin Hidden Markov Model (ChromHMM) track [59] in the UCSC Genome Browser [60] (Fig. 4D grey highlighted region).

To investigate G4 formation in living cells, we performed permanganate/S1 nuclease footprinting with direct adapter ligation and sequencing (PDAL-seq) in HEK293 cells [61, 62]. This cell line was chosen due to its extensive annotation in the Encyclopedia of DNA Elements (ENCODE) database [63]. We also included data of G4 ChIP-seq data using the BG4 antibody [64] and assessed key histone modifications (H3K4me1, H3K4me3, H3K27ac) and TF binding from ENCODE datasets in HEK293 cells. PDAL-seq is a footprinting method that utilizes the ability of S1 nuclease to cleave single-stranded DNA regions at G4 motifs, resulting in a characteristic footprint – an absence of sequencing reads at the G4 site with elevated coverage in the flanking regions as shown for two replicates in Fig. 4D. PDAL-seq replicate 1 showed a more pronounced footprint slightly shifted upstream of the G4-SNV compared to replicate 2. To validate G4 formation identified by PDAL-seq, we incorporated BG4-ChIP-seq data (resulting in sequencing peaks in regions of G4 formation), which correlated with the detected PDAL-seq footprints. A second G4 cluster identified by PDAL- and BG4 ChIP-seq G4 signals was located further upstream within an alternative *MICB* promoter.

Although the target region did not exhibit H3K4me3, we observed moderate H3K27ac and bimodal H3K4me1 enrichment (no H3K4me1 signal directly at the G4-SNV, but up- and downstream signals) – marks associated with active enhancers. The predicted TF binding site from JASPAR (Table 2) was confirmed by slightly downstream KLF1 and SP3 ChIP-seq signals. Minor MAZ binding was detected directly at the G4-SNV region. KLF7 was also found in this region, albeit this was not predicted by JASPAR.

Chromosome conformation capture (Hi-C) data obtained from the UCSC Genome Browser further confirmed physical long-range interactions between the G4-SNV region and the upstream promoter region enriched with H3K27ac, H3K4me3, KLF10, MAZ and SP3 signals. Moreover, liver-derived Hi-C data revealed robust chromatin contacts between this enhancer region and two loci, one upstream of the *MICB-DT* gene (hg38; chr6: 31,479,587) and the second downstream of *MICB* (hg38; chr6: 31,519,587; Additional file 2: Fig. S13). Notably, the upstream position at chr6: 31,479,587 is annotated as enhancer region in UCSC genome browser [60].

CD spectra ranging from 220–340 nm confirmed the formation of a parallel G4 structure for the G4 motif for both alleles – the non-effect allele C and effect allele T – with a characteristic negative peak at 240 nm and a positive peak at 260 nm in a buffer containing 50 mM Tris and 150 mM KCl (Fig. 4E). The slight shoulder at 290–300 nm indicated the presence of a second G4 topology, probably of hybrid conformation. As control for CD spectra, we have used a G4 motif located in the *MYC* promoter region [49] forming a K^+^-dependent parallel G4 structure (Additional file 2: Fig. S14). To infer changes in the G4 stability, melting curve analyses were performed at 260 nm across a temperature range of 24 °C to 92 °C using two different buffer concentrations: 50 mM Tris combined with either 50 mM or 150 mM KCl. Both buffer conditions revealed a difference in the melting points of the two alleles. While for 50 mM KCl, the motif with the C-allele showed a lower stability compared to the T-allele (ΔTm = 5.7 °C, *p* = 2.942 × 10^–6^, Welch’s t-test, Fig. 4F), for 150 mM the trend was reversed with the T-allele showing a lower stability than the C-allele (ΔTm = 9.4 °C, *p* = 0.014, Welch’s t-test, Fig. 4F). Notably, with 150 mM KCl the T-allele showed negative values for the melting curve, which probably indicates not only a change in stability, but also in the conformation. Overall, the SNV affects the stability of the G4 structure, as reflected by significant differences in melting temperatures, although the direction of the effect depends on the K⁺ concentration.

Since the G4 structure is located on the template strand, the different stabilities of G4 motifs harboring either the non-effect allele C or the effect allele T (corresponding to G and A in the G4 motif), could influence gene expression by presenting barriers for RNA polymerase II with different stabilities. To detect the regulatory function of the G4, we cloned the G4 motifs upstream of the luciferase gene on the template strand in the same orientation as found in the human genome. The pqsfinder-predicted motif is slightly shorter than the G4Hunter motif, with three bases (AAG) less on the 3’ end (pqsfinder: Fig. 4G and G4Hunter: Additional file 2: Fig. S15A). Therefore, also the predicted stems and loops were slightly different. The reporter assay confirmed a 2.7-fold increase in promoter activity by the non-effect allele C in HEK293 cells. The destabilizing effect allele T led to a 3.3-fold enhanced promoter activity. The difference between the effect and non-effect alleles did not reach statistical significance (Fig. 4G).

**Fig. 5:**
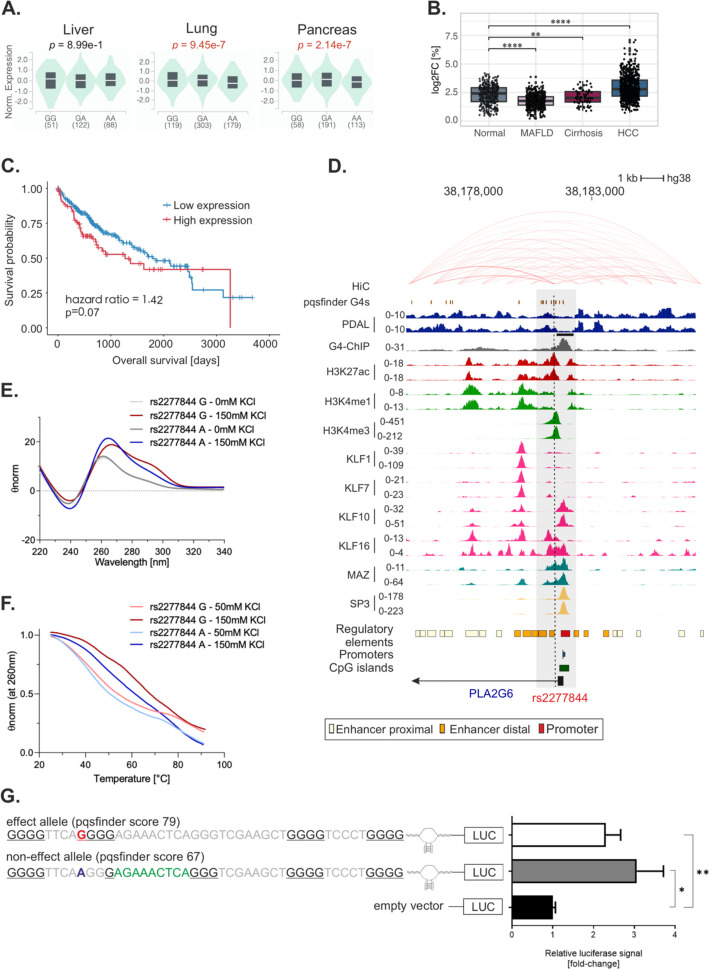
Molecular characterization of the rs2277844 G/A G4-SNV in intron 1 of *PLA2G6*. **A** *PLA2G6* expression correlated with the genotype of the G4-SNV rs2277844. The effect allele G (stabilizing variant) is associated with increased expression in lung and in pancreas tissue. Data were obtained from the GTEx database [54]. *p*-values were adjusted for multiple testing using false discovery rate correction. **B** *PLA2G6* expression decreased in metabolic-associated fatty liver disease (MAFLD) but then increased in more severe disease stages until the formation of hepatocellular carcinoma (HCC). Data were obtained from GepLiver database [65]. Wilcoxon rank sum test with BH-adjusted *p*-values.** C** Elevated *PLA2G6* expression was associated with lower overall survival, albeit only borderline significant. Data were obtained from TCGA [57]. A log-rank test was used to assess statistical significance. **D** The G4-SNV was detected slightly upstream of a PDAL-seq footprint (blue) as well as a BG4-ChIP-seq peak (grey) [64] in HEK293 cells. Enrichment of H3K27ac (red) and H3K4me3 (green), but not with H3K4me1 was observed, consistent with promoter activity. The G4-SNV is located within an annotated regulatory region as depicted by the UCSC-annotated enhancer-proximal region [60] (dark orange rectangles). KLF16 and MAZ binding was detected at the G4-SNV region, while KLF10 and SP3 peaks appeared further downstream overlapping the canonical promoter region of *PLA2G6*. **E** CD spectra of the effect allele (**G**) and the non-effect allele (**A**) demonstrated parallel G4 formation in a K.^+^ concentration-dependent manner. The effect allele G caused a slight topological change to a hybrid structure, visible as an increased peak at 295 nm. **F** CD melting curve analyses showed a slight increase in stability for the effect allele (**G**) versus the non-effect allele (**A**) with a ΔTm of 3.0 °C (*p* = 0.133, Welch’s t-test) for the 150 mM KCl buffer. The G4 structure is probably not fully assembled in the 50 mM KCl buffer, indicated by the shape of the melting curve lacking the plateau at temperatures below 30 °C. **G** The G4-motif containing either the effect (**G**) or the non-effect allele (**A**), was cloned upstream of the luciferase gene on the template (non-coding) strand as found in *PLA2G6*. The underlined bases represent the bases of the G-tract, grey bases are found in loop regions and green bases mark bulges. The G4-SNV is visible in red (**C**) and blue (T). G4 motifs and their scores were predicted by pqsfinder. Luciferase reporter assays in HEK293 cells revealed a 3.0-fold increase in promoter activity of the G4 including the non-effect allele (**A**) compared to the empty vector, while the effect allele G caused a 2.3-fold increase in promoter function (Welch’s t-test). **** *p* < 0.0001, ** *p* < 0.01 and * *p* < 0.05

The second G4-SNV analyzed in detail is the stabilizing rs2277844 G > A variant in *PLA2G6*, with the G allele being identified as a MetD-protective allele. The effect allele (G) promotes G4 stabilization and is also located on the template strand with the gene being encoded on the minus strand. Allele frequencies for *PLA2G6* of individuals with European ancestry (sample size 14,286) are G = 0.429/A = 0.57 according to dbSNP of the NCBI database [56]. GTEx data showed that the GG genotype was associated with higher expression compared to the destabilizing AA genotype in lung and pancreas, but not in liver tissue (Fig. 5A). Moreover, while MAFLD patients showed decreased expression of *PLA2G6*, it increased during the progression to the more severe cirrhosis stage and finally to HCC (Fig. 5B). The correlation of overall survival and *PLA2G6* expression levels differed between cancer types. While the overall survival was reduced in the high expression group in HCC patients (Fig. 5C), the opposite was observed for lung and pancreatic cancer patients, suggesting a cancer-type dependent role of *PLA2G6* (Additional file 2: Fig. S16A. lung and B. pancreas).

PDAL-seq and G4 ChIP-seq signals were detected slightly downstream of the G4-SNV overlapping the annotated promoter region from UCSC. Nevertheless, PDAL-seq (especially of replicate 1) and G4 ChIP-seq signals still coincide with the G4-SNV indicating its formation.

The G4-SNV colocalized with active histone marks H3K27ac and H3K4me3. Although the G4-SNV is located adjacent to an annotated enhancer-proximal region, the absence of H3K4me1 marks directly at the G4-SNV suggests that this region does not function as an enhancer in HEK293 cells; instead, the two upstream elements overlapping H3K4me1 marks are more likely to carry enhancer activity. This is consistent with the very low Hi-C signals at the G4-SNV, although several contacts with regulatory regions were observed, including an enhancer element within the *PLA2G6* gene body (hg 38, chr22: 38,161,507; Additional file 2: Fig. S17). Therefore, this element may be active in other cell types. Although not predicted by JASPAR [43], KLF16 and MAZ binding was observed directly at the G4-SNV region, and KL10 and SP3 were detected downstream at the canonical *PLA2G6* promoter element (Fig. 5D).

CD spectra revealed that the non-effect allele (A) forms a parallel topology, while the effect allele (G) showed a shift towards a hybrid G4 structure depicted by an increased peak shoulder at 295 nm, suggesting a change in G4 topology (Fig. 5E). Melting curve analyses revealed slight differences in G4 stability under both buffer conditions (50 mM KCl and 150 mM KCl). Of note, with the 50 mM KCl buffer both G4 oligos are probably not fully folded or only partially assembled. This is reflected in the shape of the melting curve, since no plateau was observed even at temperatures below 30 °C [66]. Within the 150 mM KCl buffer, the effect allele (G) exhibited increased stability, although this was not statistically significant (ΔTm = 3 °C, p = 0.133, Welch’s t-test, Fig. 5F), which is consistent with the predictions by pqsfinder and G4Hunter.

Next, we performed a luciferase reporter assay with the *PLA2G6* G4 motif placed on the template strand upstream of the luciferase reporter gene, in the same orientation as it is found in the human genome. The G4 motif for *PLA2G6* was differently predicted by pqsfinder and G4Hunter (Additional file 2: Fig. S15B). We observed a significant 3.0-fold increased promoter activity by the non-effect allele (A), and only a 2.3-fold increased promoter activity of the more stable effect-allele (G) in comparison to the empty vector (Fig. 5G). The difference between effect and non-effect allele was not significant.

Both reporter assays – for *MICB* and *PLA2G6* – showed that G4 structures upstream of the luciferase gene, even on the template strand, can act as promoters, while their stability has a fine-tuning effect on the amount of the transcript produced. In conclusion, this multi-layered analysis, combining GWAS data, expression data, structural validation, histone marks, TF binding, and chromatin conformation data strongly supports that the target G4-SNVs in *MICB* intron 1 and *PLA2G6* intron 1 reside within functional elements corresponding to cryptic promoters previously identified by Demircioglu and colleagues [32].

### AlphaFold 3 predicts structural changes induced by the effect alleles

Having demonstrated that effect alleles can alter the structural properties of G4 motifs, we next sought to explore whether AlphaFold 3 [67] could predict genotype-specific changes in DNA structure. AlphaFold 3 has already been used to predict G4 structures in agreement with experimental data [68, 69]. Thus, to assess the potential of this approach, we selected a subset of G4-SNVs and modeled their sequence-specific conformations. Specifically, we analyzed the structural impact of three stabilizing variants (rs2277844, rs9556760, rs8103500) and four destabilizing variants (rs4318227, rs2855804, rs4516268, rs7203846), including G4-SNVs located in *MICB* (rs2855804) and *PLA2G6* (rs2277844). These variants were chosen from Table 2 (genotype-specific changes; first three G4-SNVs) and Additional file 1: Table S16 (four additional candidates). This exploratory approach allowed us to examine whether AlphaFold 3 can model subtle allele-dependent shifts in G4 structure topology and stability. Although the models frequently captured many of the non-canonical interactions expected in G4 formation, model quality was generally at best moderate (predicted Template Modeling (pTM) score ≤ 0.5, Additional file 2: Fig. S18A). This was in accordance with observations made for other G4 conformations and points to the modeling challenges posed by DNA and RNA aptamers [69]. For the analyzed set of G4-SNV sequences, pTM scores were on average slightly higher in effect alleles as compared to the non-effect alleles. Notably, substitutions of single nucleotides show (sometimes strong) effects on conformation, as seen from the RMSD between models (Additional file 2: Fig. S18B). The highest pTM score was observed for rs2277844 (*PLA2G6*), which was also classified as a stabilizing variant. All other G4-SNVs showed pTM scores below 0.5. Among these, rs9556760 was likewise identified as a stabilizing variant, while rs4318227 and rs4516268 were consistent with predictions as destabilizing variants. Interestingly, rs2855804 and rs8103500 displayed effects opposite to those predicted by pqsfinder and G4Hunter, but this should be interpreted with caution due to the low pTM scores for these G4-SNVs.

Additional file 2: Fig. S19 shows two examples of the conformational effects of single nucleotide substitutions on G4s predicted by AlphaFold 3. In both cases, structural changes of the backbone are remarkably high, as reflected by RMSD values of 6.09 Å (rs2277844) and 9.25 Å (rs2855804), while only minor changes had been observed for rs2277844, and none for rs2855804 by CD experiments. Also, all models recapitulate the expected central arrangement of potassium ions, surrounded by stacked guanines. It has to be noted, however, that both global and local confidence metrics suggest low model accuracy, thus caution is required in their interpretation.

## Discussion

GWAS have identified numerous SNVs linked to complex traits and diseases. However, the translation of these associations into biological mechanisms remains a major bottleneck. DNA secondary structures, particularly G4s, have emerged as important regulatory elements enriched in promoters [16, 18] and enhancers [17, 19], where they can modulate transcription in manifold – often context dependent – ways either by serving as binding hubs for transcription factors [4, 5], assisting in higher order chromatin folding [70], acting as nucleosome organizers [18] or presenting barriers for the RNA polymerase [71]. To investigate a potential overlap between SNVs associated with metabolic diseases and changes in G4 formation, stability and topology, we (i) intersected GWAS findings from a cohort including 1,252,787 individuals of European ancestry [27] with G4 structures predicted by pqsfinder [34] and G4Hunter [35, 36], (ii) assessed the effect of risk and protective alleles on G4 stability and (iii) performed detailed analyses about their genomic annotation, the overlap with transcription factor binding sites as well as their co-occurrence with alternative promoter elements identified across several tumor entities [32]; and analyzed the regulatory potential of two target G4-SNVs in cancer-associated alternative promoter elements of *MICB* and *PLA2G6* in detail.

### G > A/C/T substitutions predominantly occur in G4 motifs and cause genotype-specific changes in G4 stability

Approximately 1.22% of the analyzed SNVs overlapped with predicted G4 motifs, with G4Hunter detecting a slightly higher proportion (1.55%) than pqsfinder (0.90%). These differences highlight the variability in sensitivity and search criteria between the two prediction tools. In comparison, Gong et al. analyzed all variants from the dbSNV database build 154 (excluding indels) [25] and identified approximately 5 million out of more than 640 million SNVs that cause gains, losses, or structural conversions of G4s, representing roughly 0.8% of SNVs located within G4 regions [25]. We analyzed 618,106 SNVs and observed a slightly higher co-occurrence with G4s, possibly due to our focus on disease-associated SNVs.

The non-random overlap of SNVs with G4 motifs predicted by both tools aligns well with the overall elevated mutation frequency detected for non-canonical DNA structures [41, 72, 73]. This can partly be attributed to their ability to impede polymerase progression during replication, resulting in fork stalling and DNA double-strand breaks that ultimately promote mutagenesis at G4 structures [74, 75]. Furthermore, the co-occurrence of G4s with RNA:DNA hybrids (R-loops) during transcription has also been proposed as a driver of mutations at non-canonical DNA structures [76, 77]. In line with observations of Guiblet et al. [41] and Li et al. [78], we found slightly more SNVs located in G4 loops compared to stems. Elevated GC content has been shown to predict increased substitution rates [42]. Nevertheless, G4 stems are inherently richer in guanines than loops, and the overall GC content at most loop positions of G4s harboring a MetD-associated SNV was not significantly different from the randomly sampled control G4 dataset, at least for pqsfinder-predicted G4s. This is in line with recent findings by Richl and colleagues investigating cancer-associated SNVs [79]. The observation that the first position of all three loops shows the lowest GC content overlaps well with the most abundant G4 type with a single adenine-loop. This, also, is in good accordance with the observation of GGGA and GGGT repeats being highly abundant in transposable and SVA elements [21, 75]. Therefore, we can exclude GC content alone as the driving force for the elevated loop substitution frequency. Instead, this pattern likely results from the greater accessibility of these regions, making them more prone to mutagenesis than the structurally constrained G-tracts within stems. Such flexibility may be advantageous in regulatory regions that must adapt to dynamic transcriptional needs, as changes in loops can modulate G4 topology or stability, potentially influencing transcription factor binding and downstream regulation.

When analyzing the types of nucleotide changes, we found that G > A and to a lesser extent G > C/T substitutions were significantly enriched at G4s compared to SNVs not located in G4 motifs, without a significant difference in G4 occurence on the plus or minus strand. G > A transitions are amongst the most common substitutions in the human genome [80] which may partly explain their elevated frequency within G4s. However, the high stacking of guanines also increases their susceptibility to oxidative damage, notably the formation of 8-oxo-guanine (8OG) [68, 81]. C > T substitutions are frequently observed on the complementary strand of 8OG sites in both patient samples and cells deficient in the OGG1 and MUTYH repair enzymes [82]. This elevated formation of DNA adducts potentially contributes to the increased rate of G > A substitutions observed within G4-forming sequences. A second mechanism which may contribute to the elevated G > A substitution rate involves the deamination of methylated cytosines, which results in C > T transitions, and correspondingly G > A on the complementary strand. Although G4s are typically found in unmethylated regions [39, 83, 84], loss of DNA methylation – commonly observed within gene bodies or certain promoters during tumorigenesis – can promote G4 formation [62], and thus probably also the activation of alternative promoters [31, 32]. Elevated C > T (G > A) substitution rates have been detected as signature for loss of methylation across different tumor types [85].

The observed substitution pattern also aligns with the reduction in G4 stability caused by the effect allele, regardless of whether the SNV was classified as risk or protective variant. In a scenario, where the G > A variant occurs within a stem, a loss in stability can be observed by the disruption of the G4 stem. G4s with short central loops tend to adopt a parallel topology, whereas those with longer central loops more likely fold into antiparallel or hybrid conformations [8, 75, 86–88]. We hypothesize that the tendency for marginally lower stability in effect allele G4s is related to their presumed role in gene regulation. It has been shown previously that regulatory element G4s are typically formed by sequences conserved across related species [89], that the structures themselves are relatively stable, and under negative selection, much more so in the stems than in the loops [78, 89]. The stability, however, is not always the highest possible but rather is modulated by other forces. Owing to the fact that G4s are not the only components of these regulatory regions, they have to coexist with TFBS and other functional sequences and often the genes they control are members of larger functional networks. Accordingly, it has been observed that different regulatory networks employ G4s of differing stability [90]. Perhaps, if there is some kind of tunable regulation in a gene regulated via G4s, the G4s must not be rigid structures (only resolved by e.g., a helicase). Instead, they should be structures that can dynamically respond to the presence or absence – or even the concentration – of an upstream regulatory agent or molecule, acting as a fine-tuning mechanism rather than a simple on/off switch.

### G4-SNVs overlap with metabolic disease associated transcription factors

We observed a high enrichment of G4-SNVs in intronic regions, which is largely attributable to the general high abundance of SNVs in intronic and intergenic regions, and only 12.6% of the analyzed SNVs occur in promoter regions. In contrast, G4 structures are typically more frequent in promoters, with 29.75% of pqsfinder- and 24.46% of G4Hunter-predicted G4s falling within promoter regions (defined as ±3 kb of the transcription start site).

The G4-SNV datasets showed highly similar TFBS enrichment patterns, characterized by a high abundance of ZnF proteins. SP5 and MAZ were among the most enriched transcription factors. When comparing motifs carrying the effect versus non-effect allele, VEZF1, SP5, KLF4, and MAZ consistently ranked among the top altered binding sites for both tools. Although SP5 has not yet been described as G4-binding TF, it belongs to a large family of C2H2-type zinc finger proteins, with members known to bind to G4s including SP1, KLFs and MAZ [4, 5, 44, 91]. Zinc-finger proteins in general have been associated with G4s in human, chimp and mouse promoter regions [92], and SP and KLF transcription factors have been shown to play an important role in liver metabolism and insulin signaling [93]. SP5 is a target gene of the WNT/β-catenin signaling pathway [94, 95], which plays a central role in lipid and insulin metabolism and is critically implicated in the development of insulin resistance and inflammation, both hallmarks of metabolic diseases [96, 97]. In line with this, MAZ is overexpressed in liver cancer, where it contributes to nuclear phase separation and is preferentially located in transcriptionally active compartments, often recruited through G4 structures [44]. The top significantly changed transcription factor binding site in the pqsfinder G4-SNV dataset was PDX1, known as an important driver of development of different forms of diabetes [45]. PDX1 plays a critical role during the transcription of the insulin gene and has been shown to be absent in cells exposed to long-term hyperglycemia (glucose toxicity), a condition linked to oxidative stress as observed in type 2 diabetes [98, 99]. Whether PDX1 is able to bind to G4 structures remains to be elucidated.

The link to aberrant transcription factor binding is further supported by the Gene Ontology analysis, which revealed that genes overlapping with G4-SNVs are enriched in functional categories related to transcription factor binding, histone-modifying activity, and transcriptional processes. Notably, several enriched terms were related to calcium metabolism and cadherin binding, aligning with the known role of impaired calcium signaling in metabolic disorders [46].

### Destabilizing *MICB* and stabilizing *PLA2G6* G4-SNVs are located in regulatory elements and overlap with cancer-associated alternative promoters

*MICB* encodes for stress-inducible surface molecules that activates natural killer (NK) cells as well as two subpopulations of T-cells – CD8+ T and γδT-cells – through the activating NK group 2D (NKG2D) receptor [100, 101]. Expression of *MICB* is induced during cellular stress, including inflammation and cancer [58]. Membrane-bound *MICB* is recognized by NKG2D on NK cells, triggering immune-mediated cell killing [100]. However, in cancer, *MICB* expression is often dysregulated: cells can shed MICB through interaction with the ERp5 chaperone and cleavage by ADAM10, ADAM17 and MMP14, releasing soluble MICB. This circulating form leads to NKG2D receptor downregulation and impaired NK function, thereby promoting tumor immune evasion [37, 102, 103]. Serum levels of soluble MICB are significantly elevated in patients with chronic liver disease and hepatocellular carcinoma compared to healthy individuals [104]. Therefore, individuals carrying the risk allele of rs2855804 likely exhibit elevated *MICB* expression and may already display deregulated NK cell signaling.

Increased *MICB* expression correlates with the allele at the alternative G4-promoter variant rs2855804 [54], which is associated with disease and occurs at higher frequencies in patients with metabolic disorders [27], and expression levels also steadily increase with disease progression from MAFLD to HCC [65]. High *MICB* transcript and protein levels are also observed in metabolic-associated steatohepatitis (MASH) — correlating with disease severity markers such as hepatocyte apoptosis and fibrosis [105] — and with advanced cancer stage and metastasis [106].

The investigated MetD-associated *MICB* G4-SNV resides within a 5-tetrad G4 structure containing two bulges and is located on the template strand in a G4-dense region. Destabilization of this G4 by the T allele may facilitate RNA polymerase II progression, thereby enhancing transcription, similar to G > A substitutions in the *MYC* promoter that disrupt a silencer G4 and promote transcription [107]. The luciferase assay confirmed a similar genotype-specific regulatory effect. Nevertheless, the binding of transcription factors in the vicinity of G4-SNV also points toward transcription factor–mediated regulation of gene expression, likely influenced by the G4-SNV’s effect on G4 stability. Although KLF1 has been shown to act as erythroid-specific transcription factor important during the differentiation and maturation of erythroid cells [93, 108], it is also implicated in macrophages of the fetal liver [109]. Furthermore, KLF1 downregulation in cervical cancer cell lines has been shown to inhibit metastasis and invasion of cervical cancer cells via inhibiting the PI3K/Akt signaling pathway [110]. KLF1 primarily functions as a transcriptional activator [111], though it can also act as a repressor in certain contexts [112]. KLF7 is linked to obesity and type II diabetes [113] and it is involved in many different cancer types (for review see [114]). SP3 acts as dose-dependent transcriptional repressor of *ABCA1*, *LCAT*, and *CETP* by competing with SP1 for binding in the promoter elements and SP3 mutant mice show decreased fetal liver mass [93]. MAZ is known as G4-binding protein [44] as well as a transcriptional repressor in the *c-MYC* P2 promoter element [115].

An identical G4 motif was detected within the intragenic region of the *MICA* gene, suggesting a conserved regulatory element within the *MICA/MICB* cluster. The duplication of *MICA* lineage II (LII) likely gave rise to *MICB* [116], explaining the shared G4 sequence. Both genes are highly polymorphic, a diversity thought to result from evolution, as new variants may help prevent viral immune evasion [101]. Thus, studying mutation frequencies in G4 motifs during viral infections could offer insights into host–virus coevolution and immune adaptation as well as the relation of virus-associated cancers and G4 motifs.

The second investigated G4-SNV is located in *PLA2G6,* which plays an oncogenic role in melanoma and hepatocellular carcinoma by up-regulating cell proliferation, migration, invasion and down-regulating cell apoptosis and changes in the lipid metabolism [37, 117]. During liver carcinogenesis, *PLA2G6* initially increases during the inflammatory process until the development of HCC in a mouse model [118], similar as observed in humans, with *PLA2G6* being a direct target of c-MYC, a transcriptional driver of HCC. Of note, a drop in *PLA2G6* expression has been detected in the later cancer stages with poorly differentiated HCC in mice [118], which could also be the case in humans and would be in line with the observation that overall survival was lower for the groups expressing low levels of *PLA2G6* for lung and pancreas cancer.

The stabilizing *PLA2G6* rs2277844 G/A G4-SNV introduces a fourth G (instead of three Gs) in the second stem. This change is accompanied by lower expression for the AA genotype compared to the GG genotype in lung and pancreas, where low expression of *PLA2G6* is linked to overall decreased survival. Results from the allele-specific luciferase reporter assay showed higher expression for the non-effect allele A compared to the effect allele G, illustrating the allele-specific regulatory activity of this element, albeit not agreeing with the patients’ data from GTEX database data for lung and pancreas. In general, reporter assays have to be taken with care, since they are artificial systems and cannot fully recapitulate the complex interplay between transcription factors and G4 structures. Key regulatory features such as chromatin architecture and DNA methylation are absent in these assays. However, they remain valuable experiments to trace down effects on gene expression caused by G4 motifs differing by as little as a single nucleotide.

Moreover, for rs2277844 G/A, CD spectroscopy revealed a subtle shift in G4 topology from a parallel to a hybrid conformation, which may account for the observed reduction in expression. The regulatory potential of this region is further underlined by the overlap with H3K4me3 and H3K27ac histone marks as well as KLF16 and MAZ transcription factor binding.

Taken together, these two examples of G4-SNVs found in cancer-associated alternative promoter elements link MetDs also with and elevated cancer risk via the overlap with G4 motifs. As shown by Esnault and colleagues, destabilization of G4s leads to changes in transcriptional activity, since G4s per se function as promoter elements [18] – as shown also by our reporter assays. Genotype-specific differences in our promoter assays were not significant in both genes, nevertheless the G4 structures showed a significant increase in promoter activity when tested in a plasmid lacking a minimal promoter. Although we have investigated only two genes in more detail, in luciferase assays these two show a genotype dependent-expression pattern and both genes are differentially expressed in different tissues as well as during the progression of metabolic-associated fatty liver disease to hepatocellular carcinoma.

## Conclusions

To address methodological variability, we compared two distinct G4 prediction tools, which use different algorithms, to assess SNVs associated with MetDs overlapping with G4 forming sequences. Despite inherent differences in the algorithms, both tools consistently showed similar patterns. One major limitation of our study is the lack of knockdown or overexpression experiments for the transcription factors investigated in *MICB* and *PLA2G6*. Nevertheless, our detailed analysis supports the view that, in addition to G4 structures acting as barriers to polymerase progression during transcription, MetD-associated SNVs within G4 motifs can also alter transcription factor binding and act as alternative promoters modulated by MetD-associated SNVs.

In summary, our analysis revealed that a subset of metabolic disease-associated SNVs colocalize with G4 motifs, influencing their stability and topology. These structural alterations caused by the patients’ genetic background can modulate transcription factor binding, chromatin accessibility, and the utilization of promoters or enhancers, ultimately driving changes in gene expression patterns and potentially contributing to the elevated cancer risk observed in metabolic disease patients. Our findings support the growing evidence that G4s represent a critical regulatory layer similar to epigenetic marks and may mediate genetic risk in complex diseases.

## Methods

### Aim of the study

The aim of this study was to investigate if SNVs associated with metabolic syndrome overlap with predicted G4 forming motifs, with a focus on their potential impact on G4 stability or topology changes, by comparing two different G4 prediction tools, pqsfinder [34] and G4Hunter [35, 36]. We further examined whether the identified G4-SNVs are enriched in specific genomic regions, co-localize with transcription factor binding sites, whether these binding sites are affected by substitutions and thereby changed, and as novel approach if the detected G4-SNVs are co-occurring with cancer-associated alternative promoter elements.

### SNV dataset

We utilized the dataset of Park et al. investigating 1,252,787 individuals of European ancestry, accessible through the EMBL-EBI GWAS catalog [119] accessible with the ID GCST90444488 [27, 120]. All SNVs with a Benjamini Hochberg (BH) corrected *p* < 0.05 were included, resulting in a set of 618,106 SNVs. At a given locus, the effect allele is the variant associated with an increase (risk effect) or decrease (protective effect) in the trait, with the effect size reported as beta value (β). To infer if a difference occurs in the overlap with G4 motifs, we analyzed positively- (risk) and negatively- (protective) associated SNVs separately. The non-effect allele is the allele at the same locus that is not associated with the reported effect.

### Prediction of G4 motifs

G4 motifs were predicted for the human genome assembly GRCh38 using pqsfinder [34] and G4Hunter [35, 36] algorithms. G4 annotations of pqsfinder-predicted G4 motifs and their stability scores were accessed from ([121], https://pqsfinder.fi.muni.cz/genomes) using a minimal stability score threshold of 47, with all other parameters used as default values. To generate bed files with annotations for G4Hunter-predicted G4 motifs, the GRCh38 reference genome was downloaded from the UCSC genome browser [60]. G4s were predicted using the G4HunterDetect function provided by the G4SNVHunter package [36]. For G4 detection the default parameters of 1.5 for the absolute G4Hunter score threshold and a window size of 25 were applied.

### Calculation of G4 scores for effect and non-effect alleles

The reference sequences for the predicted G4s were obtained using the GRCh38 reference genome and the bedtools getfasta function [122]. The MetD SNV table included both the effect allele and the non-effect allele. The “effect” sequence was defined as the allele (risk or protective) associated with MetDs in the GWAS, whereas the “non-effect” sequence corresponded to the allele not associated with MetDs. The corresponding G4 sequences and single-nucleotide variants (SNVs) can be found in the “Mutated_G4s.bed” files for both G4Hunter and pqsfinder predicted G4s. G4 scores for these sequences were predicted using the G4HunterScore function from the G4SNVHunter package [36], while pqsfinder scores were calculated using the score function from the pqsfinder R package [34]. For G4s which pqsfinder did not consider as a G4 motif anymore after mutation, the pqsfinder score was set to 0.

### Detection of G4–SNV overlaps

Overlaps between SNVs and predicted G4 motifs were identified using the bedtools intersect function [122]. Annotation to genomic regions was performed with the ChIPseeker package [123] in R [124]. To evaluate whether the overlap between SNVs and G4 motifs occurs randomly, we generated a randomized background dataset that preserves the chromosome-wise distribution, as well as the GC content in ± 25 bp windows around the G4-associated SNVs. Specifically, we first counted the number of G4-associated SNVs per chromosome, calculated the GC content in ± 25 bp windows and binned them in 9 categories, ranging from bin 0 to bin 9 (bin 0: 0–10%, bin 2: > 10–20%, …, bin9: > 90–100% GC content) and then randomly sampled an equivalent number of SNVs from the whole 618,106 SNV dataset for each chromosome in the corresponding GC bin. Using bedtools intersect [122], we quantified the overlaps between these randomized SNV sets and G4 motifs. This procedure was repeated 1,000 times to establish a null distribution, and *p*-values were calculated using a permutation test to assess the significance of SNV enrichment within G4 motifs.

To compare if MetD-associated SNVs were overrepresented in G4s in general, the 618,106 SNV were randomly shuffled over the whole genome correcting for GC content as well as for chromosome-wise distribution as explained before and intersected with the pqsfinder and G4Hunter predicted G4 motifs using bedtools intersect [122]. This approach was repeated 1,000 times and *p*-values were calculated using a permutation test.

### Identification of canonical G4 motifs from pqsfinder- and G4Hunter- predicted G4 motifs

Canonical G4 motif detection and SNV classification were performed in R version 4.5.0 (2025–04–11) using custom scripts. The analysis pipeline identified SNVs overlapping predicted canonical G-quadruplex (G4) motifs and classified their positions within G4 stems or loops. Predicted G4-forming sequences were extracted from genomic regions containing SNVs detected by pqsfinder or G4Hunter using BED-formatted input data. Effect and non-effect sequences were screened for canonical G4 motifs defined by the regular expression pattern allowing loop lengths up to 12 nts:

(G3,)([ATCG]1,12)(G3,)([ATCG]1,12)(G3,)([ATCG]1,12)(G3,)

### Analysis of SNV occurrence in loop and stem regions

Both effect and non-effect sequences were analyzed to determine whether a canonical G4 motif was present in (i) both alleles, (ii) only the non-effect allele (motif loss), or (iii) only the effect allele (motif gain). SNVs were classified according to their location within stems, loops, or outside the canonical G4 motif window. Variants outside the defined canonical motif were excluded from downstream analyses, as such sequences would typically represent bulged or non-canonical structures not considered here.

Substitution frequencies were quantified separately for stems and loops by dividing the number of observed SNVs by the total number of analyzed nucleotides within each region (stem or loop) and significant differences were calculated by using a two-sided Benjamini–Hochberg corrected Poisson test.

### GC content analysis of canonical G4 loop regions

The GC content of G4 loop regions was computed from canonical motifs as described above. For each loop, the GC fraction was calculated at every nucleotide position. First a control dataset of G4 motifs not overlapping with MetD-associated SNVs was prepared for both tools individually. To evaluate whether G4s containing SNVs differ in loop GC composition, an equal number of canonical G4s as observed in the pqsfinder- or G4Hunter G4-SNV dataset were randomly drawn 1,000 times from the full control dataset to generate a bootstrap background distribution. Mean GC fractions and 95% confidence intervals (CIs) were derived for each position. The same workflow was used for the G4-SNV dataset (without random resampling) to calculate the GC content for each position of each loop.

### G4-SNV substitution direction and strand bias analysis

We compared the base substitution patterns of G4-SNVs located within predicted G-quadruplex (G4) motifs across the plus and minus DNA strands, distinguishing between risk-associated and protective variants based on GWAS β-values.

Substitutions were classified by substitution type (e.g., C > T, G > A) and strand orientation. Separate mutation spectra were then computed for G4s on the plus and minus strands and for a GC content–matched control SNV dataset located outside predicted G4 regions with an adjusted GC content of > 0.40 (lowest GC content of a G4 motifs in G4Hunter and pqsfinder datasets). Base substitution frequencies were normalized as proportions of total variants. Strand asymmetry was further quantified as the difference in substitution frequency (Δ ratio = G4⁺ – G4⁻).

To examine G4 orientation relative to gene transcription, G4-SNVs were annotated with gene information using the TxDb.Hsapiens.UCSC.hg38.knownGene [125] package and ChIPseeker [123] package in R [124]. Each G4-SNV was classified as being on the template or non-template strand. The distance of G4-SNVs to transcription start sites (TSS) was analyzed by calculating strand-specific density distributions (± 1 Mb window) to assess positional enrichment of G4-SNVs relative to the TSS.

### Identification of overlaps between transcription factors and G4-SNVs

To identify overlaps of G4-SNVs with transcription factors and their binding sites, G4 sequences containing the effect and non-effect alleles were overlapped with transcription factor binding motifs from JASPAR (version 2024)[43] using R version 4.4.1 [124] and R Studio version (2023.09.1) [126]. Annotation to genomic regions was performed with the ChIPseeker package [123].

### Gene Ontology (GO) enrichment analysis of genes near G4-SNVs

Gene Ontology (GO) enrichment analysis was performed to identify molecular functions associated with genes located near G4-SNVs. The analysis was conducted in R version 4.4.1 [124] and R Studio version (2023.09.1) [126] using the clusterProfiler [127] and annotatr [128] packages. GO enrichment of Molecular Function (MF) terms was performed using the enrichGO() function from clusterProfiler (OrgDb = org.Hs.eg.db, ontology = “MF”, pAdjustMethod = “BH”, q-value < 0.05). Rich factor values were computed for each term as the ratio of significantly enriched genes to the total number of genes annotated for that term.

### Find overlaps with cancer-associated alternative promoters

To examine whether G4-SNVs overlap with alternative promoter elements, we extracted alternative promoter annotation from the publication of Demircioglu et al. [32]. The alternative promoter position is given as 1 bp position reflecting the transcription start site of the alternative isoform. To investigate whether G4-SNVs are in their proximity, GenomicRanges objects [129] elongated by ± 250 bp windows from the transcription start sites were generated and intersected with the G4-SNV positions. To focus on these G4s with G4 motifs with the greatest effect on G4 stability, we applied thresholds of |Δpqsfinder score|= effect-allele score – non-effect-allele score > 10 and |ΔG4Hunter score|> 0.1 (Additional file 2: Fig. S11).

### Extraction of gene expression datasets

Gene expression data for genotype specific transcription patterns were downloaded from the *Genotype-Tissue Expression* (GTEx) project [54] for liver, lung and pancreas. In addition, we extracted gene expression data for different phenotypes such as metabolic-associated liver disease (MAFLD), cirrhosis and hepatocellular carcinoma (HCC) from the GepLiver database [65] using all existing expression data from *The Cancer Genome Atlas* [57], GTEx [54], *Gene Expression Omnibus* (GEO) [130] for normal controls, metabolic- associated fatty liver disease (MAFLD, also called non-alcoholic fatty liver disease (NAFLD)), cirrhosis and hepatocellular carcinoma (HCC).

### Survival analysis

Survival data for 361 liver hepatocellular carcinoma (LIHC), 504 lung adenocarcinoma and 177 pancreas cancer patients were retrieved from cBioportal [131] using TCGA data [57]. Patients were stratified by their gene-specific *MICB/PLA2G6* mRNA expression z-scores into quartiles, and significant differences in overall survival rates were assessed by log-rank sum tests. For Fig. 4C and Fig. 5C, we displayed only the samples from the lowest and highest expression quartiles for LIHC.

### Non-B DNA detection with permanganate/S1 nuclease footprinting with direct adapter ligation and sequencing (PDAL-seq)

HEK293 kidney cells were selected to assess G4 formation due to the extensive availability of datasets covering various epigenetic marks within the ENCODE database [63]. PDAL-seq was performed according to the protocol described in [61, 62]. In short, 5–7 × 10^6^ cells were incubated with 40 mM KMnO_4_ stock solution followed by library preparation and sequenced on a NovaSeq platform with approximately 80 million reads. After demultiplexing the raw reads and quality control, reads were aligned to the human reference genome hg38 using BWA-MEM [132]. Subsequent filtering removed low-quality reads (mapping quality MQ < 30). BamCoverage files in bigwig format were generated with a 50 bp bin size and normalized to 1 × coverage and visualized in integrative genomics viewer (IGV) [133]. After creating histogram files from bigwig files using bedtools bamCoverage [122], the histograms were inverted by dividing each read by the number of total reads. This results in peaks instead of valleys. This file then was used for peak calling using SICER [134]. Detailed analysis information can be found in [61].

To compare PDAL-seq with G4 chromatin immunoprecipitation and sequencing (ChIP-seq) data, we downloaded BG4 ChIP-seq data (SRR14879746, [135]) and IgG negative control (SRR14879747, [136]) in HEK293 cells from the Gene Expression Omnibus (GEO) database [130] from Li et al. [64] and aligned them to the human genome assembly version GRCh38 with BWA-MEM [132]. BamCoverage files (bin size 50, normalized to 1 × coverage) were displayed in IGV [133].

### Histone mark, transcription factor binding and non-B DNA formation datasets

H3K27me3, H3K27ac and H3K4me1/3 histone marks and KLF1 [137], KLF7 [138], KLF16 [139], SP3 [140] and MAZ [141] transcription factor ChIP-seq data were obtained from ENCODE [63] for HEK293 cells and visualized in IGV [133]. Furthermore, we extracted the HiC annotation track from H1-hESC within the UCSC genome browser [60] as well as from the 3D genome browser [142] using the liver dataset of Leung et al. with 10 kb resolution [143, 144]. Additional file 1: Table S18 lists all analyzed datasets.

### Circular dichroism spectroscopy

Circular dichroism (CD) spectra were obtained from single stranded DNA oligos containing the sequence from the predicted G4 motifs with the effect and non-effect alleles (Additional file 2: Methods Sect. 1.1). All oligos were reconstituted in 50 mM Tris–HCl (pH 7.5) and either in 0 mM, 50 mM or 150 mM KCl in a final volume of 200 µL with an oligo concentration of 5 µM. As control we have used an oligonucleotide containing the sequence from a promoter-G4 in *MYC* forming a parallel G4 structure [62]. CD spectra and melting profiles were recorded using the Chirascan™ Plus CD Spectrophotometer instrument (Applied Photophysics, Leatherhead, United Kingdom) equipped with a Peltier temperature-controlled cuvette holder and in-cuvette temperature sensor in a 0.5-mm path length quartz cuvette. Spectra were scanned from 340 to 220 nm with a step size of 0.2 nm and a bandwidth of 1 nm at 25 °C. Four spectra per sample were averaged. After blank correction, the CD ellipticity θ in millidegrees was normalized using the relation θnorm = θ/(32,980 × c × l), where c is the DNA concentration in mol/L and l is the path length in cm.

CD spectra were smoothed using locally weighted scatterplot smoothing (LOWESS) in GraphPad Prism (version 8; San Diego, CA, USA). Ellipticity values (θnorm, mdeg) were plotted against wavelength (nm), and a non-parametric LOWESS fit was applied to minimize spectral noise and highlight local trends without distorting the overall shape of the spectrum. The default smoothing factor was used. The resulting smoothed curves were used for visualization.

### Melting curve analyses

Melting curve analyses were performed at 260 nm from 24–92 °C in four replicates (temperature setting time: 200 s, step: 1 °C, scan time per point: 0.5 s). Data points were blank corrected, normalized to 24 °C, visualized and fitted with a sigmoidal curve using the default parameters in GraphPad Prism (version 8; San Diego, CA, USA).

### AlphaFold 3 predictions

#### Structure modeling and model confidence

AlphaFold 3 [67] was used to generate 3D structure models of seven G4 effect sequences and their non-effect counterparts. We selected three G4-SNVs overlapping with alternative promoter elements that exhibit genotype-dependent expression, as shown in Table 2. Additionally, we included four G4-SNVs from Additional file 1: Table S16 that – while not showing genotype-dependent expression changes – also overlap with alternative promoters and are identified by both G4Hunter and pqsfinder. Models were calculated for single-stranded DNA molecules, with five potassium ions included. Global model confidence was assessed with the predicted template modeling (pTM) score obtained from AlphaFold 3 and categorized as high (≥ 0.7), moderate (0.5 − 0.7), or low (< 0.5). For local model confidence, AlphaFold’s predicted local distance difference template (pLDDT) scores were used, in categories of high (> 70), moderate (50–70), or low (< 50).

#### Model similarity and molecular graphics

Model analysis and molecular graphics were performed with UCSF ChimeraX [145]. Model similarity was quantified with pairwise root-mean-square deviation (RMSD) values calculated over all equivalent backbone C4' atoms.

### Luciferase reporter assay

To verify the regulatory role of identified G4-SNVs, the G4 regions were cloned into pCpG-free vectors (Invivogen, France) without a minimal promoter. Short single stranded complementary G4 oligos harboring restriction enzyme cut sites for NsiI and HindIII on the 5’ and 3’ sites respectively were mixed equimolar and heated to 95 °C and slowly cooled to 20 °C over a duration of 1 h in a thermocycler to get double stranded fragments (Sigma Aldrich, Austria, Additional file 2: Methods Sect. 1.2). After double digest of 500 ng G4 fragments and plasmid with NsiI and HindIII (NEB, Austria) at 37 °C for 20 min, digested insert fragments and the plasmid were extracted using the ReliaPrep DNA extraction and cleanup kit (Promega, Austria). DNA concentrations were determined using a Qubit fluorometer. Ligation was performed with T4 DNA ligase (NEB, Austria) at a 1:5 plasmid-to-insert molar ratio, using 40 ng starting material of plasmid DNA in a final reaction volume of 20 µl at 16 °C overnight.

5 µl of the ligated construct was transfected to chemically competent *E.coli* GT115 cells (*pir* mutant strain, InvivoGen, France) and spread on Luria–Bertani (LB) plates containing zeocin (25 µg/ml; InvivoGen, France). Single colonies were picked and incubated in 3 ml LB-zeocin medium overnight. Plasmid was extracted with PureYield Plasmid Midiprep System (Promega, Austria) and successful cloning was verified by Sanger sequencing (Microsynth, Austria, Additional file 2: Methods Sect. 1.2.2).

24 h prior to transfection, 0.5 × 10^5^ HEK293 cells per well were seeded in 24 well plates with RPMI medium + 10% fetal bovine serum without antibiotics. 500 ng plasmid (containing lucia/gaussia luciferase, InvivoGen, France) and 25 ng pGL4 SV40 background plasmid (firefly luciferase, Promega, Austria) were transfected for 24 h using Lipofectamin 3000 reagent (Thermo Scientific, Austria). After cell lysis, chemiluminescent read-out was detected on a Tecan Spark plate reader (Tecan, Austria). Lucia readouts were normalized to the firefly luciferase (background signal) and finally, data were expressed as fold-change normalized to the empty vector.

### Statistical analysis

All statistical analyses were performed using R [124] and R Studio [126] or GraphPad Prism (version 8; San Diego, CA, USA).

#### Generation of a random SNV dataset and permutation test

We generated 1,000 randomized SNV datasets by sampling SNVs chromosome-wise to match the observed counts per chromosome GC content corrected. For each random set, we recalculated the overlap with G4 regions. The empirical *p*-value was calculated as the proportion of random overlaps equal to or greater than the observed overlap, indicating significant enrichment of SNVs within G4 regions.

#### Overlap or risk versus protective SNVs with G4s

To assess whether risk and protective SNVs differ in their overlap with G4s, a Fisher’s exact test was performed.

#### Substitution directions of G4-SNVs and non-G4 SNVs

To assess whether substitution directions differed significantly between G4^+^ (G4s on the plus strand), G4^−^ (G4 motifs on the minus strand) including SNVs and non-G4-SNVs, we performed Fisher’s exact test with BH correction.

#### Loop GC content analysis

Empirical *p*-values were calculated by comparing the observed GC of SNV-associated G4s to the bootstrap distribution and adjusted for using Benjamini Hochberg multiple testing correction (BH < 0.05). Positions with significant deviations were highlighted in grey in the resulting plots.

#### Template versus non-template strand occurrence of G4-SNVs

Strand-specific enrichment of G4-SNVs was evaluated across cumulative distance windows from 0 to 1,000 kb relative to the TSS using binomial tests with the null hypothesis of equal distribution across strands. *P*-values were BH corrected for multiple testing.

#### Difference in transcription factor binding site overlaps

Significant differences in transcription factor binding site overlaps between the G4 motifs containing the effect-allele versus the non-effect allele was assessed by using a Fisher’s exact test with BH correction.

#### Genotype-specific expression

False discovery rate (FDR)–corrected *p*-values were obtained from GTEx expression Quantitative Trait Loci (eQTL) tables, as described in [30].

#### Expression in different phenotypes

Significant differences in gene expression across phenotypes were assessed using the Wilcoxon rank-sum test.

#### Survival curves

Differences in overall survival were assessed using log-rank tests.

#### Melting temperature of CD spectra

Melt temperature differences were evaluated using Welch’s two-sample t-test of four replicates.

#### Luciferase reporter assays

*P*-values were calculated using Welch’s two-sample t-test, based on three biological replicates with four technical replicates each.

## Supplementary Information

Additional file 1: Contains Supplementary Tables S1-S18.

Additional file 2: Contains Supplementary Methods and figures Figs. S1-S19.

## Data Availability

Source code and analysis scripts are publicly available under the MIT No Attribution License at Github (https://github.com/angelikaLahn/MetD_G4_SNVs) [146] and Zenodo [147]. The SNV dataset was obtained from the EMBL-EBI GWAS catalog [119] with the entry ID GCST90444488 [27, 120]. PDAL-seq data were deposited under the BioProject number PRJNA1088738 with replicate 1 SRR38746355 [148] and replicate 2 SRR38746354 [149]. G4 ChIP-seq data using the BG4 antibody were downloaded from the SRA database under the BioProject number PRJNA739982 with G4-ChIP-rep2 SRR14879746 [64, 135] and G4-ChIP-IgG control SRR14879747 [64, 136]. The following data from HEK293 cells deposited in the GEO database [130] as well as the ENCODE database [63] from the BioProject PRJNA63443 were use for analysis: H3K4ac (GSE174866) [63, 150], H3K4me1 (GSE174861) [63, 151], H3K4me3 (GSM945288) [63, 152], KLF1 (GSE92104) [63, 137], KLF7 (GSE91907) [63, 138], KLF10 (GSE127337) [63, 153], KLF16 (GSE105645) [63, 139], MAZ (GSE91636) [63, 141], SP3 (GSE91528) [63, 140]. HiC data of the STL011 liver dataset (GSM1419086) [143, 144] with the BioProject number PRJNA253910. All analyzed datasets are listed in Additional file 1: Table S18. Gene expression data were obtain through GepLiver database [65] using samples from The Cancer Genome Atlas [57], GTEx [54], Gene Expression Omnibus (GEO) [130]. Survival data were obtained via cBioportal [131] using TCGA data [57].
