# Supplementary material for: Metabolic disease variants rewire gene regulation through disruption of DNA G-quadruplex structures
Source: Genome Biol. 2026 Jun 10;27:255. doi: 10.1186/s13059-026-04147-2 (PMC13479703; doi:10.1186/s13059-026-04147-2)
Supplement: Supplementary file 1 [file 13059_2026_4147_MOESM2_ESM.docx]

**Additional file 2:**

**Metabolic disease variants rewire gene regulation through disruption of DNA G-quadruplex structures**

Angelika Lahnsteiner^1,2,3*^, Victoria Ellmer^1,2^, Esther Schönauer^2,4^, Julia Huber^1,2,3^, Matej Lexa^5^, Markus Wiederstein^6^, Angela Risch^1,2,3^

^1^Division of Cancer (Epi-)Genetics, Department of Biosciences and Medical Biology, University Salzburg, 5020 Salzburg, Austria

^2^Center for Tumor Biology and Immunology (CTBI), 5020 Salzburg, Austria

^3^Cancer Cluster Salzburg, 5020 Salzburg, Austria

^4^Division of Structural Biology, Department of Biosciences and Medical Biology, University Salzburg, 5020 Salzburg, Austria

^5^Faculty of Informatics, Masaryk University, Botanická 68a, 60200 Brno, Czech Republic

^6^Division of Protein Bioinformatics, Department of Biosciences and Medical Biology, University of Salzburg, 5020 Salzburg, Austria

*Corresponding author

angelika.lahnsteiner@plus.ac.at

University of Salzburg

Hellbrunnerstrasse 34

5020 Salzburg

Austria

**Content**

[1.	Additional methods section	2](#_Toc230676928)

[2.	Additional figures	3](#_Toc230676929)

[3.	References	16](#_Toc230676930)

# 1. Additional methods section

1.1. **CD spectra oligos**

| **SNP ID** | **Oligo** | **length [bp]** | **c[µM]** |
| --- | --- | --- | --- |
| rs2855804_T | AGGGGTCTGGAGGGGAGTGGGATGGAGGG | 29 | 5,00 |
| rs2855804_C | GGGGGTCTGGAGGGGAGTGGGATGGAGGG | 29 | 5,00 |
| rs2277844_G | GGGGTTCAGGGGAGAAACTCAGGGTCGAAGCTGGGGTCCCTGGGG | 45 | 5,00 |
| rs2277844_A | GGGGTTCAAGGGAGAAACTCAGGGTCGAAGCTGGGGTCCCTGGGG | 45 | 5,00 |
| MYC | GGCCGACTCAGAGGAGGGGTGGAAGGGGTGGGAGGGGTGGGAGG  GGTATTCGCGGGGAGGGCCCAAGGG | 69 | 5,00 |

1.2. **Luciferase reporter assay**
     1.2.1. **Oligos**

NsiI and HindIII restriction enzyme recognition sites are given in blue. The SNV positions are indicated in red.

| **SNP ID** | **Oligo** |
| --- | --- |
| rs2855804_T_top | tcagATGCATCCCTCCATCCCACTCCCCTCCAGACCCC**C**AAGCTTtact |
| rs2855804_T_bottom | agtaAAGCTTGGGGGTCTGGAGGGGAGTGGGATGGAGGGATGCATctga |
| rs2855804_C_top | tcagATGCATCCCTCCATCCCACTCCCCTCCAGACCCC**T**AAGCTTtact |
| rs2855804_C_bottom | agtaAAGCTTAGGGGTCTGGAGGGGAGTGGGATGGAGGGATGCATctga |
| rs2277844_G_top | tcagATGCATCCCCAGGGACCCCAGCTTCGACCCTGAGTTTCTCCC**C**T  GAACCCCAAGCTTtact |
| rs2277844_G_ bottom | agtaAAGCTTGGGGTTCAGGGGAGAAACTCAGGGTCGAAGCTGGG  GTCCCTGGGGATGCATctga |
| rs2277844_A_ top | tcagATGCATCCCCAGGGACCCCAGCTTCGACCCTGAGTTTCTCCC**T**TG  AACCCCAAGCTTtact |
| rs2277844_A_ bottom | agtaAAGCTTGGGGTTCAAGGGAGAAACTCAGGGTCGAAGCTGGGG  TCCCTGGGGATGCATctga |

1.2.2. **Sanger sequencing results**

**Sequencing result of *MICB* C/T – G4:**

**Sequencing result of *PLA2G6* G/A – G4:**

# 2. Additional figures

**Fig S1. G4 motif detection.** Bar plot showing shared and unique G4-SNVs identified by G4Hunter [1] and pqsfinder [2].

**Fig S2. GC content of MetD-associated SNVs in ±25 bp windows. A.** Distribution of 618,106 SNVs across GC-content bins calculated within ±25 bp windows centered around the SNVs. Bins: 1, 0–10%; 2, >10–20%; 3, >20–30%; 4, >30–40%; 5, >40–50%; 6, >50–60%; 7, >60–70%; 8, >70–80%; 9, >80–90% GC content. **B.** Shows significant differences in nucleotide composition within the ±25 bp windows surrounding the SNVs.

**Fig S3. MetD-associated SNV enrichment in predicted G4s.** The observed overlap of SNVs with pqsfinder- **(A.)** and G4Hunter- **(B.)** predicted G4s is significantly higher compared to a randomly shuffled SNV dataset corrected for SNV density at individual chromosomes as well as GC-content. Statistical significance of differences between regions was assessed using a permutation test with 1,000 iterations.

**Fig S4. Length-normalized substitution frequencies.** Substitutions identified within stems and loops of canonical G4 motifs were normalized to the total analyzed nucleotide length given in base pairs [bp]. Statistical significance of differences between regions was assessed using a Benjamini-Hochberg (BH) corrected Fisher´s exact test.

**Fig S5. Loop and stem length distribution.** Length distribution of G4 stems and loops for pqsfinder- (left) and G4Hunter- (right) predicted canonical G4 motifs are shown. Note, that the outliers in stem length result from the search algorithm (G3,)([ATCG]1,12), which did not limit the maximal number of G4 stem nucleotides.

**Fig S6. GC content of individual loop positions 1-12 across all loops of canonical G4 motifs. A.-B.** Mean GC content (lines) ± 95% confidence intervals (shaded areas) across loop positions are shown for canonical G4s identified by (A). pqsfinder and (B.) G4Hunter. GC content of G4s harboring MetD-associated G4s is indicated by black dots. Grey shading areas mark loop positions where the GC content is significantly different from the background (BH corrected *p* < 0.05).

**Fig S7. Top 20 overrepresented motifs in pqsfinder and G4Hunter canonical G4 motifs.**

**Fi S8. Strand-specific differences in G4-SNV substitution direction. A.-B.** Bar plots show the differences in the proportion of SNV directions between the positive and minus strands for G4-SNVs identified by pqsfinder (A.) and G4Hunter (B.). Results are shown separately for risk (left) and protective (right) variants. Each bar represents the difference in proportion [%] for a given SNV substitution type. Positive values indicate a higher proportion of SNVs on the positive strand, while negative values indicate enrichment on the minus strand. No significant difference was detected for +/- strand orientation for each position as indicated in Supplement Table 11 using a BH corrected Fisher´s exact test.

**Fig S9. Strand-specific distribution of G4-SNV in relation to the transcription start site. A.-B.** Density plots show G4-SNVs on the template (red) and non-template strand (blue) in relation to the nearest transcription start site (TSS). Results are shown for G4-SNVs predicted by pqsfinder (A.) and G4Hunter (B.). Distances are measured in kilobases (kb), with 0 representing the TSS position.

**Fig S10. Genomic annotation of G4 motifs and SNVs. A.-B.** Shows the annotation of G4s predicted by pqsfinder [2] (A.) and by G4Hunter [1] (B.). **C.** Shows the annotation of the MetD-associated SNV dataset used for the analysis [3].

**Fig S11. Density plots of stability scores for G4 motifs harboring MetD-associated SNVs. A.-B.** Density plots showing the differences in G4 stability scores between effect and non-effect alleles for G4 motifs containing MetD-associated SNVs for pqsfinder (A) and G4Hunter (B). Positive scores indicate stabilization, while negative scores indicate destabilization. Grey dashed lines represent the minimum thresholds of the selected G4s used in the alternative promoter overlap analysis.

**Fig S12. Alternative promoters in MICB.** The MICB genomic region is shown in the UCSC Genome Browser [4] using the hg38 chromosome assembly. G4 motifs predicted by pqsfinder [2] are indicated as black lines. rs2855804 is marked as grey line and indicated by an arrow. Transcript isoforms, UCSC-annotated promoters (light blue rectangles), and CpG islands (green rectangles) are also displayed. Additionally, regulatory elements from ENCODE (cCREs) [5] are shown, including promoters (red rectangles), proximal enhancers (yellow rectangles), and distal enhancers (orange rectangles). Alternative promoters identified by Demircioglu et al. [6] are depicted as black dashed lines.

**Fig S13. HiC contact points of *MICB* rs2855804 G4-SNP.** HiC read peaks were detected for position chr6: 31,479,587 and chr6:31,519,587 and visualized in 3D Genome Browser [7].

**Fig S14. Circular dichroism spectra for MYC control region.** KCl-dependent G4 formation is shown for the *MYC* control region [8] in buffers containing either 0 mM or 150 mM KCl.

**Fig S15. G4Hunter-predicted G4 motifs in *MICB* and *PLA2G6*.** Predicted G4 motifs and their corresponding G4Hunter scores are shown for *MICB* (A) and *PLA2G6* (B). Red and blue bases indicate the SNVs, while underlined bases mark the predicted G4 stems (shown in reverse complement orientation for *PLA2G6*).

**Fig S16. Overall survival correlating with *PLA2G6* expression. A.-B.** Kaplan Meier plots show significant reduced overall survival of lung (A.) and pancreas (B.) cancer patients with low expression of *PLA2G6*. Patients were stratified into quartile-based groups based on mRNA expression z-scores, with group sizes (n) reflecting the corresponding number of patients. Log rank test was used to assess significance. Data were extracted from cBioportal [9] using The Cancer Genome Atlas (TCGA) datasets [10].

**Fig S17. HiC data for *PLA2G6.*** HiC read peaks were detected and visualized in 3D Genome Browser [7] for position chr22: 38,161,507 (dotted line) for rs2277844, albeit the signal intensity was very low.

**Fig S18. Model characteristics of AlphaFold 3 predictions. A.** Distributions of confidence (pTM) scores of structure models generated by AlphaFold 3 for G4 motifs harboring effect (left) and non-effect (right) alleles. **B.** Structure similarity of model pairs (effect/non-effect) depending on the difference in their pqsfinder scores.

**Fig S19. Structure models as predicted by AlphaFold 3. A.** AlphaFold predictions of G4 motifs harboring effect alleles (dim grey) superimposed with their non-effect counterparts (light grey). In **B**. the same structures are shown spread apart, with colors indicating local model confidence (AlphaFold pLDDT score). Potassium ions are depicted as spheres in all models.

# 3. References

1.	Bedrat, A., L. Lacroix, and J.L. Mergny, *Re-evaluation of G-quadruplex propensity with G4Hunter.* Nucleic Acids Res, 2016. **44**(4): p. 1746-59.

2.	Hon, J., et al., *pqsfinder: an exhaustive and imperfection-tolerant search tool for potential quadruplex-forming sequences in R.* Bioinformatics, 2017. **33**(21): p. 3373-3379.

3.	Park, S., et al., *Multivariate genomic analysis of 5 million people elucidates the genetic architecture of shared components of the metabolic syndrome.* Nat Genet, 2024. **56**(11): p. 2380-2391.

4.	Perez, G., et al., *The UCSC Genome Browser database: 2025 update.* Nucleic Acids Res, 2025. **53**(D1): p. D1243-D1249.

5.	Ernst, J. and M. Kellis, *Chromatin-state discovery and genome annotation with ChromHMM.* Nat Protoc, 2017. **12**(12): p. 2478-2492.

6.	Demircioglu, D., et al., *A Pan-cancer Transcriptome Analysis Reveals Pervasive Regulation through Alternative Promoters.* Cell, 2019. **178**(6): p. 1465-1477 e17.

7.	Wang, Y., et al., *The 3D Genome Browser: a web-based browser for visualizing 3D genome organization and long-range chromatin interactions.* Genome Biol, 2018. **19**(1): p. 151.

8.	Lahnsteiner, A., et al., *G-quadruplex forming regions in GCK and TM6SF2 are targets for differential DNA methylation in metabolic disease and hepatocellular carcinoma patients.* Sci Rep, 2024. **14**(1): p. 20215.

9.	Cerami, E., et al., *The cBio cancer genomics portal: an open platform for exploring multidimensional cancer genomics data.* Cancer Discov, 2012. **2**(5): p. 401-4.

10.	Cancer Genome Atlas Research, N., et al., *The Cancer Genome Atlas Pan-Cancer analysis project.* Nat Genet, 2013. **45**(10): p. 1113-20.
